# Putative past, present, and future spatial distributions of deep-sea coral and sponge microbiomes revealed by predictive models

**DOI:** 10.1093/ismeco/ycae142

**Published:** 2024-11-15

**Authors:** Kathrin Busch, Francisco Javier Murillo, Camille Lirette, Zeliang Wang, Ellen Kenchington

**Affiliations:** Department of Fisheries and Oceans Canada, Bedford Institute of Oceanography, Dartmouth, NS B2Y 4A2, Canada; Department of Fisheries and Oceans Canada, Bedford Institute of Oceanography, Dartmouth, NS B2Y 4A2, Canada; Department of Fisheries and Oceans Canada, Bedford Institute of Oceanography, Dartmouth, NS B2Y 4A2, Canada; Department of Fisheries and Oceans Canada, Bedford Institute of Oceanography, Dartmouth, NS B2Y 4A2, Canada; Department of Fisheries and Oceans Canada, Bedford Institute of Oceanography, Dartmouth, NS B2Y 4A2, Canada

**Keywords:** biodiversity, deep-sea, sponge, cold-water coral, microbiome, mapping, spatio-temporal, prediction, environmental shifts, trait-based modeling, North America

## Abstract

Knowledge of spatial distribution patterns of biodiversity is key to evaluate and ensure ocean integrity and resilience. Especially for the deep ocean, where in situ monitoring requires sophisticated instruments and considerable financial investments, modeling approaches are crucial to move from scattered data points to predictive continuous maps. Those modeling approaches are commonly run on the macrobial level, but spatio-temporal predictions of host-associated microbiomes are not being targeted. This is especially problematic as previous research has highlighted that host-associated microbes may display distribution patterns that are not perfectly correlated not only with host biogeographies, but also with other factors, such as prevailing environmental conditions. We here establish a new simulation approach and present predicted spatio-temporal distribution patterns of deep-sea sponge and coral microbiomes, making use of a combination of environmental data, host data, and microbiome data. This approach allows predictions of microbiome spatio-temporal distribution patterns on scales that are currently not covered by classical sampling approaches at sea. In summary, our presented predictions allow (i) identification of microbial biodiversity hotspots in the past, present, and future, (ii) trait-based predictions to link microbial with macrobial biodiversity, and (iii) identification of shifts in microbial community composition (key taxa) across environmental gradients and shifting environmental conditions.

## Introduction

Cold-water coral reefs and deep-sea sponge grounds are found throughout the world’s ocean. They occur in diverse settings, such as continental shelves, slopes, seamounts, ridges, and fjords and provide a habitat for a myriad of associated organisms. These ecosystems are thus highly diverse systems, which play a key role in ocean services, integrity, and resilience. Symbioses between microbes and animals are considered key drivers of ecosystem properties. Deep-sea corals and sponges can harbor dense and diverse microbial communities inside their tissue, which play fundamental roles in key physiological processes, such as carbon, nitrogen, and phosphorous cycling [1, 2]. Interestingly both, deep-sea sponge [3, 4], as well as cold-water coral [5, 6] microbiomes were found to be host species-specific, and some parts of the microbiome show imprints of co-evolutionary dynamics between the host and its microbial partners. Microbial richness refers to the variety and variability of microbial life forms in a certain habitat. Deep-sea sponges were found to harbor up to 71 microbial phyla, comprising 201 bacterial classes [3]. In addition, a large-scale study of deep-sea sponge microbiomes revealed microbial alpha-diversity (i.e. richness) to be mainly constant across varying environmental conditions, while significant differences were observed in beta-diversity in most cases [3]. Microbial richness patterns have been well studied in sponges and shown to be an important feature of microbial community stability—here one particularly interesting trait is their separation into high microbial abundance (HMA) sponges and low microbial abundance (LMA) sponges; while some sponges contain dense and rich microbial consortia in their tissues, other species lack such dense and diverse communities. This status (HMA or LMA) does not only correlate with microbial abundance and richness, but also with differences in ecosystem function (e.g. in terms of their nutrient cycling capacity) of both metaorganism types. Typically, analyses of host-associated microbiomes are done by invasive sampling, subsequent lab processing for DNA extraction and *16S* ribosomal ribonucleic acid (rRNA) gene amplification (the V3-V4 region for bacteria), sequencing, and bioinformatic analyses [7, 8]. This approach comes with several disadvantages, the two most obvious ones being: (i) a reduction of vulnerable host species populations through sampling, and (ii) a limitation in sampling that provides only scattered snapshots in space and time. Modeling techniques represent powerful tools to make predictions and generate scenarios, by integrating previously generated knowledge.

Assessments and predictions of spatial distribution patterns have been conducted extensively for several deep-sea sponge [9, 10] and deep-sea coral species [11–17]. Although microbial (*16S* amplicon) data exist and the main driving factors have been identified for both deep-sea sponge [3, 18] and coral [19–21] microbiomes, those existing datasets represent only snapshots in space and time, and microbial information have not been incorporated into host distribution models. We consider the generation of continuous spatio-temporal distribution patterns as a next major research direction to answer research questions such as: “Where may biodiversity hotspots of host-associated microbiomes be found in the contemporary and future ocean?”, and subsequently, “How do predicted shifts in the future align with predicted patterns of the past?”. Additional questions are: “How do host-associated microbial communities shift across environmental gradients and how do abundances of key microbial taxa vary spatially?”, and lastly “How do patterns of host-associated microbial abundances relate to spatial niche separations and distribution patterns of the larger macrobial community?”. The aim for this study was to answer those questions for deep-water sponge and coral-associated microbiomes, as both sponges, as well as corals, represent key animal species of deep-ocean benthic communities.

We focus specifically on deep-sea habitats, as we see the biggest need to extrapolate existing data in this realm—the largest habitat on our planet. Sampling in the deep ocean is particularly challenging due to the needed technology and infrastructure, but at the same time we urgently need information about species distributions, interactions, and environmental variabilities in times of increasing ocean threats [22, 23]. We see an analogy in the study of environmental microbiomes, where only <1% of bacteria can actually be cultured in the laboratory [24, 25]. Although actual laboratory verifications are thus challenging, researchers are still able to derive highly valuable information about microbial community structure and function with the help of metagenomic and bioinformatic approaches. One strength of our study is to introduce a new conceptual perspective and integrative modeling approach for the exploration of host-associated microbiomes. We are aware of the current data limitations especially in deep-sea habitats, which increase uncertainties and decrease model predictability, but we hope that our approach can help to identify and then actively minimize those uncertainties over the upcoming years. As a starting point our study focusses on one of the currently most data-rich study areas in the deep ocean—the deep ocean along the North American east coast, spanning a wide geographic range from 31°N to 75°N. Further, this study is based on sponge and coral species which show a high abundance in that region, and for which microbiome data exists. Some species (e.g. *Vazella pourtalesii*) are known to only occur in that study region, while others appear to be more wide-spread. Overall, we see a great potential for our modeling approach to be transferable to other study organisms and regions.

## Materials and methods

**Figure 1:**
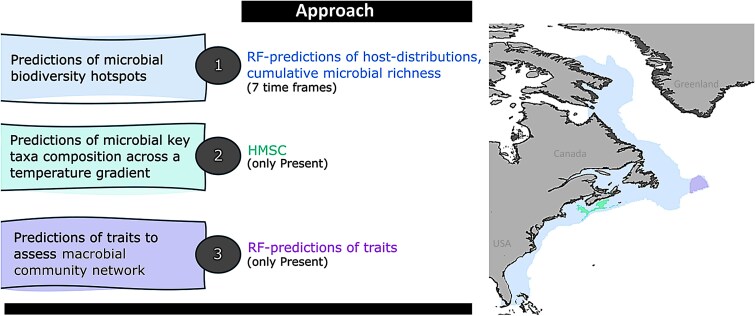
Overview of study aims, modeling approaches, and study areas for each study aim. Study areas encompass the Eastern North American shelf (blue color), the Emerald Basin and Sambro Bank (turquoise color), and the Flemish Cap (purple color). HMSC: Hierarchical modeling of species communities.

The applied workflow is described in the following and has been summarized in Fig. 1. We also provide additional figures showing workflows for individual Method sections in greater detail in the Supplementary material (see links below). The individual analysis steps, were executed in R (version 4.1.3 [26]), ArcGIS Pro (version 2.8.8 [27]), and Python (version 3.7.3 [28]).

### Spatial extent and preparation of environmental data layers for species distribution modeling

The spatial extent of this study covered an area off the North American east coast in the northwest Atlantic, spanning a large latitudinal extent from ~25°N (Nassau, Bahamas) to ~71°N (northern part of Baffin Island, Canada), (Supplementary Figs 1 and 2). In order to avoid inclusion of land points, a 5 km land buffer was applied along the coastline, and all environmental grid cells falling within this boundary were removed. The area extended seaward from the buffered coastline to the 2000 m bathymetric contour; any areas deeper than 2000 m were excluded from the study area. Raster cell size was 0.088°, using the WGS 1984 datum. With the “Calculate Geometry” tool in ArcGIS Pro the geodesic area of each grid cell was calculated, showing that grid cells at the North extended to ~31km^2^, grid cells at the south extended to ~87km^2^, and grid cells at the middle coordinate of the whole study area (~48°N) were ~64 km^2^. The study region was broken up into three different regions (A, B, C, Supplementary Fig. 1) in order to perform analysis on a sub-regional level and to allow comparability to previous studies (e.g. [10]). Region A, the northernmost subregion, spanned a geographic range from 52.1° N to 71.5°N; while region C, the southernmost subregion, spanned a geographic range from 25.0° to 40.0°. Region B was the subregion in between region A and region C.

Time-wise this study spans 214 years, from 1871 until 2085. Two static (stable in time) environmental parameters were used throughout the complete time span. These were “bottom depth”, derived from the General Bathymetric Chart of the Oceans (version 2019 [29]), and “slope”. Slope, in degrees, was derived from the bathymetry raster using the Slope Tool in ArcGIS Pro’s Spatial Analyst toolbox, after projecting the bathymetry layer to Albers projection for this analysis (and then back again to WGS 84). Bilinear interpolation was used to resample both depth and slope to match the resolution of the dynamic climatic variables (0.088°).

Dynamic (varying in time) environmental layers for our study area were derived from two sources: the Bedford Institute of Oceanography North Atlantic Model (BNAM [30]), and the Simple Ocean Data Assimilation reanalysis of ocean climate variability (SODA [31]). The complete study period was split into 7 time frames covering the years 1871–1900, 1901–1930, 1931–1960, 1961–1989, 1990–2015, 2046–2065, and 2066–2085 (Supplementary Fig. 3). The two future time frames were simulated under IPCC [32] worst-case CO_2_ emission scenarios (RCP8.5 [33]). As future time frames of BNAM were provided in delta fields, absolute values of each variable were calculated for inputting them into the species distribution models. All data derived from BNAM were calculated as averages across the complete time period of each multi-annual timeframe. Dynamic environmental layers for the present and future were mean bottom temperature, mean bottom salinity, mean bottom current velocity, mean bottom stress, mean maximum mixed layer depth, mean surface temperature, and mean surface salinity (Supplementary Fig. 4). Mean bottom current velocity (${U}_b$) was calculated from eastward seawater velocity (U) and northward seawater velocity (V), with the following formula: ${U}_b=\sqrt{\left({U}^2+{V}^2\right)}$. BNAM (and BNAM-derived) point data were used to create continuous raster surfaces in ArcGIS Pro (Point to Raster tool). All layers were displayed using WGS 84 geographic coordinates and the final raster resolution was 0.088°.

For the past, data were derived from the Carton-Giese SODA 2.2.4, 1871–2008 Assimilation Run [34], in a monthly-averaged form. Overall averages across each of the roughly 30-year timeframes were calculated. Bottom layers were calculated for mean temperature, mean salinity, mean U, and mean V with the help of the GEBCO bathymetry layer of the study area. In particular, the respective value was chosen from the SODA depth layer that was closest to the bottom depth at each grid coordinate. Mean bottom current velocity was calculated from mean U and mean V using the same formula as stated above for the BNAM data. Furthermore, using the mean bottom layer ${U}_b$, the strength of the bottom stress (${\tau}_b$) was calculated as ${\tau}_b=3.5\times{10}^{-3}\times \rho \times{U_b}^2$ for BNAM, and as ${\tau}_b=3.5\times{10}^{-3}\times \rho \times{U}_b\times \sqrt{\left({U_b}^2+0.02\right)}$ for SODA; where, $\rho$ is the density of seawater [kg m^−3^]. The density variable needed for the previous calculation was calculated with the help of the *gsw*-package in R as follows: In the first step pressure was calculated with the help of the *gsw_p_from_z* function, using depth and latitude as input variables. In the second step, absolute salinity (SA; g kg^−1^) was calculated from mean bottom salinity [psu] and pressure [dbar] (using the function *gsw_SA_from_SP*). In the third step conservative temperature (CT) was calculated from mean bottom temperature [°C], absolute salinity [g kg^−1^], and pressure [dbar] (using the function *gsw_CT_from_t*). In the final step, density was calculated from absolute salinity [g kg^−1^], CT [°C], and pressure [dbar] (using the function *gsw_rho*). Maximum mixed layer depths were calculated for each grid coordinate using the *castr*-package in R (in particular the *mld*-function), after extracting vertical depth-profiles of temperature and salinity over the complete water column at each grid coordinate, and calculating vertical density profiles following the density-calculation procedure described above. The 5 m-depth layer of SODA was taken to represent surface temperature and mean surface salinity. For all SODA layers, point data was interpolated using ordinary kriging in ArcGIS Pro, in order to match the spatial resolution (0.088°) of the BNAM layers. As the SODA data did not cover parts of the Hudson Strait/Ungava Bay, NA values were added to this area covered by BNAM in order to have the same spatial extent for both datasets. An overview containing all used environmental data layers was archived “10.17632/fx3vd2tgcf.2 (‘Data2’)”.

### Coral and sponge occurrence data

The aim of this study was to cover key coral and sponge species of the study area, which occur in high abundances, and for which information on the associated microbiome was available: the sponge *Weberella bursa*, the sponge *Stryphnus fortis*, the coral *Lophelia pertusa* (*Desmophyllum pertusum*), the coral *Desmophyllum dianthus*, and the sponge *V. pourtalesii* were found to match those criteria (Supplementary Fig. 5). A total of 11 931 raw coral and sponge occurrences were compiled for the study area. Occurrence records of *W. bursa*, *S. fortis*, *L. pertusa*, *D. dianthus*, and *V. pourtalesii* were drawn from multiple published sources ([3, 10, 17–19, 35–38]), as well as from previously unpublished data. For a more detailed overview of the individual data records used in this study, see “10.17632/fx3vd2tgcf.2 (‘Data3’)”. Total raw occurrence records compiled from these sources, were as follows: 925 *W. bursa*-related individual records (110 presences and 815 absences), 694 *S. fortis*-related individual records (69 presences, 625 absences), 2103 *L. pertusa* presence records, 3629 *D. dianthus* presence records, and 4580 *V. pourtalesii*-related records (164 presences, 4416 absences). For each coral or sponge species, raw occurrence records were filtered prior to modeling, such that only one record per grid cell was retained, and coordinates of the grid cell’s centroid were assigned to the record. If presences and absences occurred in the same grid cell, presence was used. Mapping to grid cells resulted in 99 cells with *W. bursa* presences, 55 cells with *S. fortis* presences, 142 cells with *L. pertusa* presences, 84 cells with *D. dianthus* presences, and 130 cells with *V. pourtalesii* presences (see 10.17632/fx3vd2tgcf.2  *(“Data4”)*). Cells which had recorded absences were filled with zeros. In addition, pseudo-absences were added for each species to those cells which had recorded presence-absences for any of the other coral or sponge species, assuming that the respective species would have been recorded if present. Following this procedure, all 5 coral and sponge species had records (presence + absence) in 5477 grid cells of our study area (i.e. there were records in ~15% of all grid cells).

### Random Forest predictions of five coral and sponge species occurrence

In order to predict coral and sponge distribution patterns, Random Forest (RF) models were fitted using the *randomForest*-package in R [39] with default parameters and 500 trees. Model accuracy metrics and threshold probabilities were derived using 5-fold spatial block cross-validation [40]. For each cross-validation run the threshold-independent area under the receiver operating characteristic curve (AUC) was calculated, and the mean and standard deviation were derived. The *optimal.thresholds*-function in the “PresenceAbsence” R package [41] was used to calculate several common threshold values above which a given relative probability of occurrence is considered a presence. The threshold which maximizes the sum of sensitivity and specificity (MSS) was our threshold of choice. There, sensitivity and specificity represent the proportion of accurately predicted presences and absences, respectively. Using MSS, probabilities of occurrence outcomes from each cross-validation run were converted into predicted binary outcomes which were subsequently summarized into a 2 × 2 confusion matrix. In order to assess model performance along with AUC, the sensitivity, specificity, and the true skill statistic (TSS; [42]) were derived. The *importance*-function in the R package “randomForest” was applied to evaluate the importance of the environmental predictor variables in the RF models using the mean decrease in Gini index. Functional response curves were produced to assess changes in the relative probability of occurrence across environmental variable gradients (after [43]; Supplementary Fig. 6). Here, it cannot be excluded that non-linear, non-monotonic relations between predictors and probabilities of animal occurrence are artifacts related to the nature of the underlying data, which would increase uncertainties of our model predictions.

### Computation of cumulative microbial richness

In total 99 samples were processed to analyse microbiome diversity (*16S* amplicon data). These 99 samples consisted of 16 *W. bursa*, 19 *S. fortis*, 8 *L. pertusa*, 7 *D. dianthus*, and 32 *V. pourtalesii* individuals (Supplementary Fig. 5). In addition, 17 seawater samples were analysed. The raw sequences were retrieved from the European Nucleotide Archive database and originate from [3, 18, 19] (for details on accession numbers see “10.17632/fx3vd2tgcf.2 (‘Data5’)”). As the same region of the 16S gene was amplified for all samples (V3 region), all reads were processed together within the QIIME2 environment (version 2019.10 [44]). Processing was done according to the bioinformatic pipeline published in [3]. In the present study, primers and heterogeneity spaces were removed. Then additional 15 nt were trimmed at the start of all reads, and the reads were truncated to a length of 225 nt. Chimeric reads were removed from the dataset. Amplicon sequence variants (ASVs) were generated from forward reads with the DADA2 algorithm [45]. The FastTree2 plugin was used to calculate phylogenetic trees based on resulting ASVs. Classification of representative ASVs was done with help of the Silva 132 99% OTUs 16S database [46] with the help of a *16S* region-specific trained naïve Bayes taxonomic classifier. Afterwards, chloroplast, mitochondrial, eukaryotic, and unassigned reads were removed. A sampling depth of 6000 was applied, and alpha diversity indices (Shannon indices) calculated for each of the 99 samples. Mean microbial richness estimates were calculated for all five coral and sponge species, and the seawater reference group. Cumulative microbial richness was calculated for each grid cell, by combining coral or sponge occurrence with microbial richness estimates. In particular, predictions of relative probability of animal occurrence were thresholded into a binary depiction of suitable vs. unsuitable habitat using the individual MSS values used to threshold the confusion matrices of each animal species. Afterwards these binary predictions were overlaid for all five animal species, and for those animals with a presence in the respective grid cell, mean microbial richness estimates of the respective animal species were summed up (and called “cumulative microbial richness”).

In order to assess uncertainties, standard deviations were calculated for all host species and all time frames for the RF predictions of host-species distribution patterns (via assessment of the five models used for cross validation for each host species). The mean standard deviation was calculated for each species across all seven time frames. In addition, the standard deviations were summed up across the five host species for each time frame. Uncertainties were also calculated for the microbial richness estimations. In particular the standard errors (SEs) of variations in microbial richness were calculated for each host-species. In order to check if predictions of cumulative microbial richness were higher in some geographic regions than in others, regions-wise uncertainties were calculated (here we focused only on the present timeframe): first, the standard deviations were summed up for all prevailing host species, assessed for each grid cell, and translated into relative values. Second, the SEs for all prevailing microbiomes were summed up, assessed for each grid cell, and also translated into relative values. Then both, relative host and microbiome uncertainty values were added up for each grid cell which contained a predicted microbial data point, and standardized to 100. Lastly, the medians of those values were calculated per region (Region A, Region B, Region C). In order to identify locations that require more sampling efforts to cover the full environmental gradient of the studied area with actual physical holobiont samples, grid cells that had environmental parameter values falling outside the sampled environmental envelope where pinpointed. For this, the minimal and maximal values for each of the nine environmental parameters (values for the present timeframe) were extracted for all grid cells with animal occurrence data derived from sampling. In the next step it was identified for each grid cell of the study area, how many parameters were higher or lower than their identified minima and maxima. The same procedure was done for the microbial data. In contrast to the host data, here only bottom depth, mean bottom temperature, and mean bottom salinity were considered (i.e. the most relevant parameters for microbial diversity [3]), and only those grid cells where hosts had been predicted to occur. In the last step, the number of identified parameters falling outside the sampled environmental envelope were summed up for the host and microbial analysis per grid cell. This approach was conducted for the present, and the two future timeframes, and used for plotting the summed uncertainties related to under-sampled environmental data ranges.

In order to identify shifts in microbial richness over time (present vs. future), the relative probabilities of animal occurrence projected for the two future timeframes (2046–2065, and 2066–2085) were also thresholded and cumulative microbial richnesses were calculated using the same procedure as described above. Afterwards, cumulative microbial richnesses were compared for those three time frames (present and two future timeframes), but also for the four past timeframes. The “Calculate Geometry” tool in ArcGIS Pro was used to calculate the geodesic area of each grid cell (km^2^). From there summed total areas (km^2^) were calculated of all grid cells per species for each year (Supplementary Table 1). Areas that experienced a gain, loss, or no change in cumulative microbial richness from present-day conditions were evaluated. In order to get a feeling for the magnitude of predicted shifts across the complete study area, the overall area of predicted shifts (including both, areas of gain and areas of loss) was calculated. For this, grid cells showing a predicted shift over time were summed and thereof the percentages of predicted gains and losses were calculated, respectively (Supplementary Fig. 7).

### Hierarchical Modeling of Species Communities (HMSC) to predict abundance shifts of key microbes across environmental gradients

In order to predict abundance shifts of microbes along changing environmental conditions, we focused on the microbiome of the glass sponge *V. pourtalesii*, as microbial sample numbers were largest for this species (*n* = 32). A detailed overview of the methodological workflow can be found in Supplementary Fig. 8. Based on the overall microbial community compositions, that were derived from 16S amplicon sequencing analyses (see above), “key microbial taxa” were calculated. In order to define key microbial taxa of the *V. pourtalesii* microbiome, the total microbial community was divided as follows: (i) “core ASVs” were defined as those ASVs occurring in all animals, (ii) “variable community ASVs above 10” are those ASVs occurring in ten or more animals, (iii) “variable community ASVs below 10” are those ASVs occurring in between two to nine animals, (iv) “individual ASVs” are those ASVs occurring in one animal only. In order to identify key microbial taxa, we consider ASVs that occur in ten or more *V. pourtalesii* individuals only, and which have a mean relative abundance of >1%. Following this procedure, 15 key ASVs were identified, which cover 67% of the total microbial community of *V. pourtalesii* in terms of relative abundance. After identification of key microbial taxa, our data was brought into the format needed to run the HMSC model: Relative abundances of the 15 key ASVs were standardized to 100%. The bottom temperature raster and the predicted *V. pourtalesii* occurrence raster were merged into a new raster, containing coordinates and temperature values at locations of predicted *V. pourtalesii* occurrences only (i.e. presence probabilities above the respective MSS threshold identified for *V. pourtalesii*). The study area for running HMSC models was subsetted to study region B only, as this was the area with highest predicted area shifts in cumulative microbial richness (Supplementary Fig. 7). In region B, *V. pourtalesii* is predicted to occur in a temperature range between 4.6°C and 9.2°C. Coordinates of *V. pourtalesii* individuals sampled in situ, for molecular work, were matched with the resulting temperature grid by finding the closest points of sampling coordinates in the temperature grid coordinates. Microbial communities of *V. pourtalesii* falling into the same grid cell were averaged. After doing so, four grid cells (locations) were used as input for the HMSC model. In order to enlarge the number of locations in the model, we extracted additional locations (coordinates) with the same temperature as the in situ measurements (precision of two digits °C) from the subsetted temperature grid. Our applied rational was that we expect a very similar mean microbial community composition in the same sponge species under the same temperature conditions. We thus calculated average relative microbial abundances of key ASVs per temperature category (i.e. the individual temperature values at all four sampling locations with a precision of two digits °C), and transformed them into “dummy” integers for the HMSC modeling. We then appended the data of the 18 additional locations, identified as locations with similar temperatures to the in situ sampling locations (see description above), using microbiome averages of each respective temperature category as additional data values. This procedure lead to 22 locations in total, which were used in the HMSC model as the spatial training matrix of microbiome data. We recommend to future studies that are planning to use a similar simulation approach that they should work with a larger number of real sampling locations, covering a defined environmental gradient, right from the start to avoid a usage of “dummy” integers and to potentially reach a higher predictive power for more microbial taxa in the model. We established our precise applied HMSC modeling framework through multiple test runs, in which we checked model performance indicated by the coefficient of discrimination Tjur’s R^2^ [47], which is defined as the difference between the average model prediction for successes and failures and was summarized as the mean Tjur’s R^2^ across species. A 2-fold cross-validation was performed to assess the predictive power of the model. Our final model was a HMSC which included a spatial random effect, temperature as only covariate, and that is based on a Poisson distribution. We used HMSC [48] from the “Hmsc” R-package [49] to fit a joint species distribution model on the key microbial community member level. Our response matrix were the relative microbial abundances of key microbes (transformed to dummy integers). We included a random effect at the level of sampling station using a latent factor approach [50]. After fitting the model (assuming a linear effect), we examined and ensured the convergence of the Markov chain Monte Carlo simulations and evaluated the model fit (psrf values of the beta parameters were on average 1.002 with a 95% confidence interval = 0.003). The explanatory power of the model was assessed by computing the R^2^ for each microbial species, and as mean across all microbial species. Furthermore, a 2-fold cross-validation was conducted to evaluate the predictive power of the model. Subsequently, the parameter estimates were explored and predictions made, followed by an additional step to make spatial predictions. We then standardized our spatial predictions to 100%, converting “dummy counts” into relative abundances. In the next step, we plotted our predictions across the complete temperature range, covered by the subsetted temperature grid. Based on these plots we determined temperature ranges which may have a too high uncertainty of predictability (especially those temperature ranges that were far of values with actual in situ measurements of microbial community composition). Following these evaluations, we subsetted our initial input grid to a narrower temperature range, by removing all *Vazella pourtalessii* occurrence points above or below the temperature range 6.2–8.2°C. We then reran the complete HMSC model again including all previous steps (i) set up a HMSC model with a spatial random effect and temperature as only covariate, based on a Poisson distribution, (ii) fit model, (iii) evaluate convergence, (iv) compute and show model fit, (v) show parameter estimates, (vi) make predictions, (vii) make spatial predictions, and standardize them to 100%. After these steps, we extracted the ratio of explanatory vs predictive power of all 15 key ASVs (Supplementary Table 2). We then extracted the two ASVs with the lowest positive values (ratio of 1.2 and 1.9) and plotted the relationship with temperature of those ASVs. In the next step we generated individual ASV distribution maps of those two ASVs in space. This was done for the present, and subsequently also for the two future timeframes using the predicted future host distributions of *V. pourtalesii* subsetted to the modeled temperature range of 6.2°C - 8.2°C. Furthermore, we constructed a (sparcc-) co-occurrence network based on the total microbial community in *V. pourtalesii*. Within Gephi (version 0.9) the Furchterman Reingold algorithm was used (Supplementary Fig. 9A), from there the graph object was exported to Cytoscape (version 3.10.0) and there projected into a circular layout. For the visualization only those ASVs were visualized that had a connection to at least one key ASV. “Highly interconnected” and “interconnected” ASVs were defined based on the degree of connectivity inside the network (see explanation below). ASV taxonomic identities were traced back manually. The same procedure was conducted for a subnetwork including only the connections in between “highly interconnected” ASVs. A potential correlation between network connectedness (number of edges) and model fit (explanatory power, predictive power, and ratio of explanatory power vs predictive power) was evaluated through Pearson correlation for all 15 key ASVs.

### Trait predictions of the microbial abundance status in deep-sea sponges in space

In order to perform trait predictions of the microbial abundance status in deep-sea sponges in space, we focused on the Flemish Cap area. The Flemish Cap harbors a particularly lush benthic community with sponges constituting ~95% of the total benthic biomass [51], making it a particularly suitable study site. For this approach we integrated published data of geographic sponge species distribution [52], and knowledge of the microbial abundance status of the different sponge species [3, 53] for ten sponge species (five HMA species and five LMA species; Supplementary Table 3). Here, we considered the microbial abundance status (HMA vs LMA) as a binary trait (0 absence in the grid cell, 1 presence in the grid cell), and predicted both, HMA and LMA distributions, on a continuous spatial scale with the help of two separate RF models. BNAM environmental data layers of the present timeframe, as well as slope and depth, which were snapped to the raster extend used in Murillo et al. [52], were used as environmental data layers. The RF models were run using a similar approach as described above, in brief: (i) fitting of models with the *randomForest*-package in R [39] with default parameters and 500 trees, (ii) derivation of model accuracy metrics and threshold probabilities using a 5-fold spatial block cross-validation, where for each cross-validation run the threshold- independent AUC was calculated, and the mean standard deviation were derived, (iii) extraction of the MSS, with the help of the *optimal.thresholds*-function in the “PresenceAbsence”- R package [41], and deriving sensitivity, specificity, and the TSS [42] from the confusion matrix.

We then correlated our spatial predictions of sponge microbial abundance status occurrence with predictions of overall ecosystem function, i.e. here nutrient cycling and habitat provision (derived from [52]). For this, the original rasters (created by [52]) were resampled to the 0.088 cell size using Bilinear interpolation as the resampling technique in ArcGIS Pro [10.17632/fx3vd2tgcf.2 (“Data6”)]. Afterwards, a correlation assessment between predictions of sponge microbial abundance occurrence and predictions of overall ecosystem function was done in form of Spearman correlations.

Overall biomasses of each sponge microbial abundance status were calculated, summing up the biomass values of all sponge species (published in [52]) for each of the two categories. We then computed a biomass network, integrating our newly generated information on HMA and LMA biomasses with biomass measurements of other sessile filter feeding invertebrates, which occur in high abundances at the Flemish Cap. This biomass network represents 116 different species in total, which are combined into functional (passive and active filter feeders) and taxonomic (8 phyla) groups [10.17632/fx3vd2tgcf.2 (“Data7”)]. The data in our present paper is a subset of all 285 invertebrates covered in Murillo et al., 2020 [52], based on only those organisms which fulfilled the criteria: (i) “motility” (= sessile or sessile-burrow), “degree of contagion” (= high abundance or patchy), and “feeding mode” (= active or passive filter feeding). Biomass networks (indicating correlations between biomass values of individual size groups per taxon) were calculated in R and visualized in Cytoscape (version 3.10.0).

## Results

**Figure 2:**
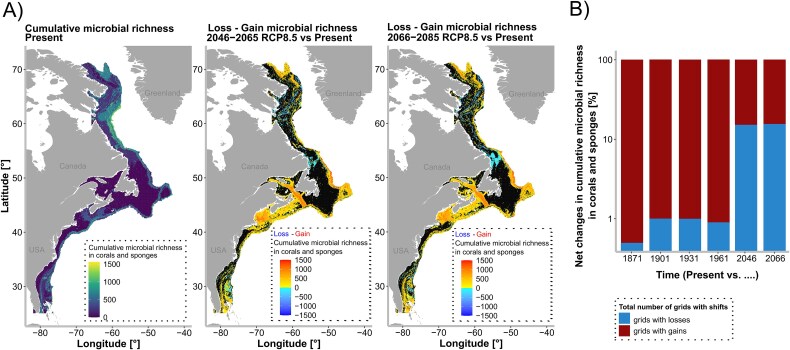
Microbial biodiversity hotspots in the past, present, and future. (A) Predictions of cumulative microbial richness in deep-sea corals and sponges for the present, as well as predicted gains and losses in cumulative microbial richness for the two future timeframes. (B) Net changes in cumulative microbial richness in corals and sponges over time, given as percentages of total shifted area. Note the logarithmic scaling of the y-axis.

Using five key species of North American east coast waters, *W. bursa*, *S. fortis*, *L. pertusa*, *D. dianthus*, and *V. pourtalesii*, putative coral- and sponge-associated microbial biodiversity hotspots were revealed in the eastern Canadian Arctic, the Flemish Cap, the Scotian Shelf, and in US waters from the border with Canada along the Eastern Seaboard to Florida (Fig. 2A). The underlying newly established simulation approach was to model the host distributions first (Supplementary Fig. 10, Supplementary Table 4), using in situ measurements and nine explanatory environmental parameters, and to then overlay the measured host species-specific microbiome data (see Materials and methods, and Supplementary Fig. 2 for more detail on the workflow). Our *16S* rRNA gene amplicon sequencing analyses revealed a mean number of observed ASVs of 161 ASVs in *W. bursa*, 480 ASVs in *S. fortis*, 149 ASVs in *L. pertusa*, 428 ASVs in *D. dianthus*, 336 ASVs in *V. pourtalesii* individuals, and 583 ASVs in seawater (see Supplementary Fig. 11). In order to provide an uncertainty estimation, standard deviations and SEs were calculated for the predictions of host species distributions, microbial richness estimates, and cumulative microbial richnesses. Cumulative uncertainties of host-species distribution predictions were ~4.5 times higher for the four past timeframes in comparison to the present and two future predictions (Supplementary Table 5). Average uncertainties of host-species distribution predictions were ~3.5 times higher for coral predictions than for sponge predictions. In particular, uncertainties were on average highest for *L. pertusa* predictions, followed in descending order by *D. dianthus*, then *W. bursa*, *S. fortis*, and *V. pourtalesii* (Supplementary Table 5). Uncertainties for mean microbial richness estimations were also higher for the two coral species in comparison to the sponge species (on average ~ 3.9 times higher). Specifically, uncertainties decreased in descending order for *D. dianthus*, *L. pertusa*, *S. fortis*, *V. pourtalesii*, and *W. bursa* (Supplementary Fig. 11). Overall relative uncertainties of cumulative microbial richness were equal for Region A and Region B (14.3 vs 14.5). Highest region-wise uncertainties of cumulative microbial richness predictions were found in Region C (~1.9 times higher than in the other regions; i.e. 27.4).

A similar approach as described above for the present, was used to make predictions under past and future environmental conditions. Strongest shifts under future projections (mainly gains in cumulative microbial richness) were predicted to occur in the Gulf of Maine and the Laurentian Channel, while losses in cumulative microbial richness in coral and sponges were predicted to occur in the Canadian Arctic. This pattern was more or less consistent for both analysed future timeframes, with differences in the intensities of predicted shifts rather than in the geographic locations of those shifts. Overall, the fraction of grid cells with observed losses in coral and sponge-associated cumulative microbial richness was predicted to increase in the future in comparison to the past (Fig. 2B). Also, the number of grid cells with at least one parameter falling outside the sampled environmental envelope increased from the present (9496 grid cells) to the future timeframes (2046–2065 timeframe: 12638 grid cells, 2066–2085 timeframe: 12814 grid cells). Throughout all three timeframes Region A had the largest number of grid cells with at least one parameter falling outside the sampled environmental envelope (mean across three timeframes = 65% of all grid cells with at least one parameter falling outside the sampled environmental envelope). Region C however had the highest overall recorded number of parameters falling outside the sampled environmental envelope per grid cell (i.e. six for hosts and microbiomes together for the future 2066–2085 timeframe; Supplementary Fig. 12).

**Figure 3:**
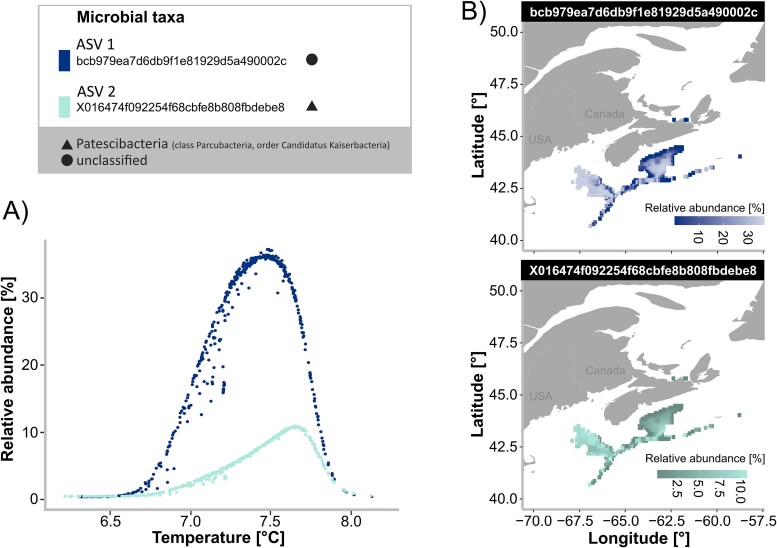
Relative abundances of key microbial taxa across shifting environmental conditions and environmental gradients. Two temperature-related key ASVs are shown by different colors, their taxonomic identity is indicated by a triangle [Patescibacteria (class Parcubacteria, order Candidatus Kaiserbacteria)], and circle (unclassified). (A) Predicted relative abundances of key ASVs with good model fit in the glass sponge *V. pourtalesii* over a fine-scale temperature gradient. (B) Predicted distribution maps of relative abundances of key ASVs with good model fit in the glass sponge *V. pourtalesii*.

Due to the observed predicted habitat shifts over time for sponges and corals, we aimed to dig deeper into modeling spatial distribution patterns of individual microbial taxa in relationship to gradients and shifts in environmental parameters. We hypothesized that relative abundances of individual key microbes in *V. pourtalesii* would change in response to gradients in environmental conditions. A similar pattern has been observed for other deep-sea sponge species at a single location with a steep local environmental gradient (i.e. on a seamount [54]), and on a basin-wide scale [3], but our goal here was to provide for the first time continuous spatial maps of key taxa relative abundances in order to get a finer understanding of high-resolution regional spatial variability of *V. pourtalesii* microbes. The *V. pourtalesii* microbiome was chosen as a study system for this approach, and we focused only on “Region B” (which includes the shelf off Nova Scotia; part of the Gulf of Maine, and areas off Newfoundland; for more details see Materials and methods and Supplementary material) for this analysis because shifts in cumulative microbial richness were predicted to be largest in this region for the two future timeframes (Supplementary Fig. 7). We identified 15 key microbial taxa (see Materials and methods for criteria on how they were identified) and we consider them to be representative of the overall *V. pourtalesii* microbial community as (i) they all together cover the majority (i.e. 67%) of the total *V. pourtalesii* microbial community in terms of relative abundances, and (ii) their taxonomic composition, also strongly resembles the community composition described in [55]. Out of these 15 key microbial taxa we identified two key microbial taxa which had a strong relationship with temperature when using our model. Taxonomically, one of the two microbial taxa belonged to the phylum Patescibacteria (class Parcubacteria, order Candidatus Kaiserbacteria), while the other microbial taxon remained unclassified. In order to detect shifts of those taxa across a very fine/continuous temperature gradient (which can also guide the design of future lab experiments), microbial key community compositions were predicted across a fine-scale temperature gradient based on a HMSC-modeling approach. We identified different optimal temperatures for the two key microbial taxa at 7.5°C for ASV1 and 7.7°C for ASV2 (for a temperature range between 6.2°C and 8.2°C), and detected slight differences in their response to changes in temperature (Fig. 3A). We focused on temperature as it has repeatedly been identified as a major driving factor of sponge microbial community composition (e.g. [3, 56]). Predicted spatial distribution maps (Fig. 3B) were then generated for the two temperature-related key microbial taxa, providing for the first time a continuous picture of how relative abundances of *V. pourtalesii*-associated microbial taxa may vary geographically. We also compared those maps for the present timeframe with extrapolated maps for the two future timeframes, and found larger areas with lower relative abundances of both ASVs for both future timeframes in comparison to the present (Supplementary Fig. 13). But how do changes in key microbial taxa translate into overall microbial community composition? In order to evaluate how predicted shifts in relative abundances of the key temperature-driven ASVs may translate into shifts in overall microbial community composition, we constructed co-occurrence networks (Supplementary Fig. 9). Those co-occurrence networks overall resembled the strong intertwining of *V. pourtalesii* key microbes: Seven of the 15 key ASVs showed network connections to a large number of additional other ASVs (and were therefore called “highly interconnected ASVs”), while eight ASVs had network connections to solely other key ASVs (and were therefore called “interconnected ASVs”; Supplementary Fig. 9A and B). The taxonomic composition of both, highly interconnected and interconnected ASVs were composed of similar taxonomic groups (Proteobacteria, Patescibacteria, and unclassified; Supplementary Fig. 9C). The number of positive and negative correlations was more or less balanced for the complete network (as well as for the subnetwork in between highly interconnected ASVs Supplementary Fig. 9D), and the two key temperature-driven ASVs split across both groups (highly interconnected ASVs and interconnected ASVs; Supplementary Fig. 9B). Lastly, the number of network connections were not significantly correlated with the model explanatory power (Pearson correlation 0.235, *p*-value = 0.400), model predictive power (Pearson correlation 0.035, *p*-value = 0.902), nor model ratio (explanatory power vs predictive power; Pearson correlation −0.007, *p*-value = 0.980).

**Figure 4:**
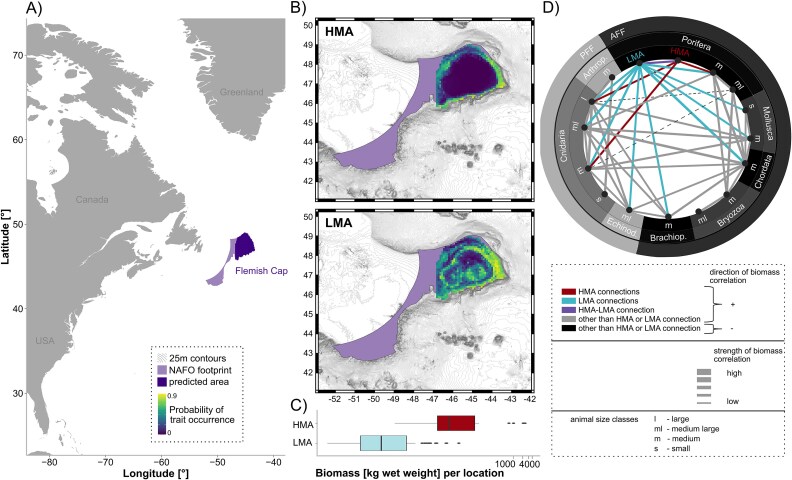
(A) Overview of study area location. The Flemish Cap is indicated by dark purple color. (B) RF-probability maps of HMA- and LMA-occurrence. (C) Comparison of biomass per location for HMA against LMA sponge species. (D) Biomass-correlation-network between HMA- and/or LMA-sponges with other filter feeding invertebrates, which occur in high abundances at the Flemish Cap. The biomass network represents 116 different species in total, which are combined into two functional groups (PFF: passive and AFF: active filter feeders), eight phyla (Porifera, Mollusca, Chordata, Bryozoa, Brachiopoda, Echinodermata, Cnidaria, Arthropoda), and four size classes (l = large, ml = medium large, m = medium, s = small). Connections of taxa with HMA sponges are colored in red, connections with LMA sponges in blue, and connections between HMA and LMA sponges in purple. Other positive correlations are colored in gray, while the only two negative connections (between medium large Porifera and medium Cnidaria, as well as large Cnidaria, respectively) are indicated by dashed lines and a black color. Positive correlations are overall stronger than negative ones (see thickness of lines; line thickness is given in simplified relative terms in the legend—not in exact values corresponding directly to the figure values).

Following predictions of cumulative microbial richness in corals and sponges, as well as predictions of individual microbial taxa and overall microbial community composition in relation to environmental shifts and gradients, we sought to explore the usability of trait-based modeling approaches to improve our understanding of macrobial community networks. Focusing on the Flemish Cap area, we assigned the HMA-LMA status to ten key sponge species, and used occurrence data of those species to predict the spatial distribution of the HMA-LMA-status at the Flemish Cap (Fig. 4A and B; Supplementary Table 6). The HMA-status was predicted to occur exclusively at the rim of the Flemish Cap and the main correlating physical parameter with this status is temperature (Supplementary Fig. 14). In contrast, occurrence of the LMA status sponges was predicted to be much more widespread through the Flemish Cap. The main correlating physical parameter of this status is salinity. However, it also has to be noted that the predictive capacity of the model was worse for LMA sponges than for HMA sponges, which may be related to a larger intra-group variability of LMA sponges. Interestingly we found a slight correlation between occurrence of the HMA status and overall predicted nutrient cycling capacity, and a stronger one between occurrence of the LMA status and overall predicted nutrient cycling capacity (both significant with *p*-value<0.001). A similar trend was observed for a correlation between each sponge microbial abundance status and overall predicted habitat provision (Supplementary Table 7). Interestingly, the clear spatial distribution pattern of HMA-occurrence seems to be driven strongly by presence of geodid sponges, and comparisons with historic data (dating back to >16.7 ka BP) suggest that this pattern has most likely existed at the Flemish Cap for a long time (Supplementary Fig. 15). Notably, although the HMA status is predicted to occur at a much narrower range in the slope waters at the Flemish Cap, biomass per location is much higher for HMA sponges than for LMA ones (Fig. 4C). A biomass network with other filter-feeding invertebrates at the Flemish Cap suggests that HMA sponge biomass is mainly correlated with cnidarians (corals) and other sponges, while biomass of LMA sponges is correlated with a much larger number of taxa (Fig. 4D).

## Discussion

This study identified putative microbial biodiversity hotspots in the past, present, and future along the North American east coast. In particular, the eastern Canadian Arctic, the Flemish Cap, the Scotian Shelf, and the US waters from the border with Canada along the Eastern Seaboard to Florida, were identified as hotspots for coral- and sponge-associated microbial biodiversity (with highest predictive uncertainties for the latter region). These regions therefore seem promising for future research efforts, which should target and integrate microbial diversity assessments of additional deep-sea benthic habitat-forming animals. The Gulf of Maine and the Laurentian Channel were identified as areas with strongest predicted shifts under future projections. While mainly gains in cumulative microbial richness were predicted for those areas, losses in cumulative microbial richness were predicted to occur in the Canadian Arctic, pointing toward a need to gain a better understanding of ecological dynamics and biological feedbacks in those two areas. Overall, our results imply that in relative terms more deep-water locations along the North American east coast will face losses in cumulative microbial richness in the future in comparison to the past. This supports previous studies showing ongoing alterations and declines in microbial diversity in diverse habitats (e.g. [57]), but it also has to be noted that our predictions reveal mostly gains in cumulative microbial richness for the future (when considering all regions and absolute terms), and that uncertainties (of host-distribution predictions) were much higher for the past time frames in comparison to the present and two future timeframes in our study. Our study shows that the here presented modeling approach seems to be overall better suited for modeling sponge-associated microbiomes than for modeling coral-associated microbiomes, with uncertainties of host-species distribution predictions and of mean microbial richness estimations being (3.5 and 3.9 times) higher for coral predictions than for sponge predictions. This suggests that the analysed deep-sea sponges may possess a stronger host-species specificity of their microbiome, and potentially a lower randomness in host-species distribution patterns. Both of these observations could potentially be explained by sponges being more evolutionarily ancient than corals [58] and by the resulting eco-evolutionary dynamics. Taking together all these just mentioned observations, our study highlights that host-associated microbial landscapes are highly dynamic in time, and that spatial areas and ecosystems on the North American east coast have faced continuous invasions and introductions of new metaorganisms (host + microbiome) over the 214 year time frame analysed.

This study pinpointed potential temperature-related shifts in microbial community composition, and derived individual geographic maps showing predicted relative abundances of key microbes across an environmental gradient. Two key microbial taxa (ASVs) with a high relative abundance and prevalence, as well as a strong temperature-relationship were picked (for more details on key microbial taxa see Materials and methods). We identified different optimal temperatures for the two key microbial taxa and detected slight differences in their response to changes in temperature. As temperature is known to critically affect many cellular processes and metabolisms, understanding the mechanistic relationships between growth optima for certain microbial taxa and temperature will be an interesting, but challenging (due to the very low cultivation success of many sponge-associated microbes) endeavor for future studies. We consider one benefit of our here applied community modeling approach (HMSC) in identifying preferences in co-occurrence and environmental conditions of individual microbial taxa. The here generated predicted spatial distribution maps provide for the first time a continuous picture of how relative abundances of *V. pourtalesii*-associated microbial taxa may vary geographically in the present and future. Taxonomically, one of the two picked key microbial taxa belonged to the phylum Patescibacteria (class Parcubacteria, order Candidatus Kaiserbacteria), while the other microbial taxon remained unclassified. Our previous work [55] aimed to identify functional strategies and metabolic networks of the main *V. pourtalesii* symbionts. There, Patescibacteria were identified as anaerobes with reduced genomes that tap into the microbial community for resources [55]. Based on our previous work we expected a strong physiological coupling of the identified key microbial taxa with other members of the *V. pourtalesii* microbiome. In order to evaluate how predicted shifts in relative abundances of the key temperature-driven ASVs may translate into shifts in overall microbial community composition, we constructed co-occurrence networks. Those co-occurrence networks suggest indeed that changes in abiotic conditions (temperature) may not only impact single key microbial taxa, but may trigger cascades through a modular network of microbial taxa. We initially hypothesized that the HMSC-model fit of temperature-dependence would decrease for ASVs with a large number of network connections. We came to this hypothesis because we would expect a “biotic buffering effect” against changing abiotic conditions upon a stronger connectedness between microbial taxa. The number of network connections were not significantly correlated with the model explanatory power, model predictive power, nor model ratio (explanatory power vs predictive power), which again suggests that changes in abiotic conditions may not only impact single microbial taxa, but indeed a larger network of taxa.

In this study we also tested the usability of trait-based modeling approaches to improve our holistic understanding of (deep-sea) benthic habitats. The underlying idea here was to assess potential niche separations of sponge holobionts based on their microbial abundance status and to get an insight into potential species interactions on a sponge ground level. The HMA-status was predicted to occur exclusively at the rim of the Flemish Cap, while the LMA status was predicted to be much more widespread throughout the Flemish Cap. Analyses of previously published spiculae data suggested that the distinct spatial distribution of the HMA-status at the rim of the Flemish Cap seems to be persistent since ancient times (dating back to >16.7 ka BP). Occurrences of both, the HMA- and LMA-status, were both significantly correlated with overall predicted nutrient cycling capacity, related to predicted habitat provision capacity, and strongly driven by temperature and salinity, respectively. Although the HMA-status was predicted to occur at a much narrower range in the slope waters at the Flemish Cap than the LMA-status, we found biomass per location to be much higher for HMA sponges than for LMA ones. HMA sponge biomass was mainly correlated with cnidarian (coral) biomasses and that of other sponges, while biomass of LMA sponges was correlated with a much larger number of taxa, including echinoderms, brachiopods, chordates, and mollusks in addition to the cnidarians and sponges. While corals, echinoderms, and other sponges, were recorded to interact with sponges through trophic interactions [59–63], co-occurrences with brachiopods, chordates, and mollusks have been recorded at the Flemish Cap [64] but are currently not understood mechanistically to the best of our knowledge. Taken together we found a spatial niche separation between HMA and LMA sponges and also different co-occurring sessile filter feeding invertebrates with each of the two sponge types (so presumably different associated food webs). We thus believe that this trait-based approach is helpful to assess biodiversity-ecosystem function relationships and microbial-macrobial interactions. Evaluating microbial-macrobial relationships through such a trait-based approach represents a promising tool to further incorporate microbial data into a larger ecosystem context and into larger food web analyses. One example for a future direction of this approach may be to evaluate the role of the associated microbiome in facilitating the host to function as a capable space competitor on unprecedented spatial scales (see also literature on space competition between sponges and corals assessed based on local sampling efforts, e.g. [65–69]).

Taken together, all our results suggest that, despite a cumulative uncertainty (added up for each modeled level of organization: the environmental predictors, the host distributions, the microbiome predictions), spatio-temporal predictions of host-associated microbial communities can reveal interesting ecological patterns, help to fill data gaps in under-sampled habitats, and may guide future experiments.

## Supplementary Material

Supplements_Busch_et_al_ycae142

## Data Availability

The data and data links of this paper were deposited in the open access Mendeley database: 10.17632/fx3vd2tgcf.2. The code was deposited in github and archived in the Zenodo database: https://doi/10.5281/zenodo.14024663.
