# Supplementary material for: Putative past, present, and future spatial distributions of deep-sea coral and sponge microbiomes revealed by predictive models
Source: ISME Commun. 2024 Nov 15;4(1):ycae142. doi: 10.1093/ismeco/ycae142 (PMC11694675; doi:10.1093/ismeco/ycae142)
Supplement: Supplements_Busch_et_al_ycae142 [file supplements_busch_et_al_ycae142.pdf]

Supplementary Material

to:

Putative past, present, and future spatial distributions of deep-sea coral and sponge microbiomes revealed by predictive models

Kathrin Busch, Francisco Javier Murillo, Camille Lirette, Zeliang Wang, Ellen Kenchington

contains:

- Supplementary Figures 1-15
- Supplementary Tables 1-7

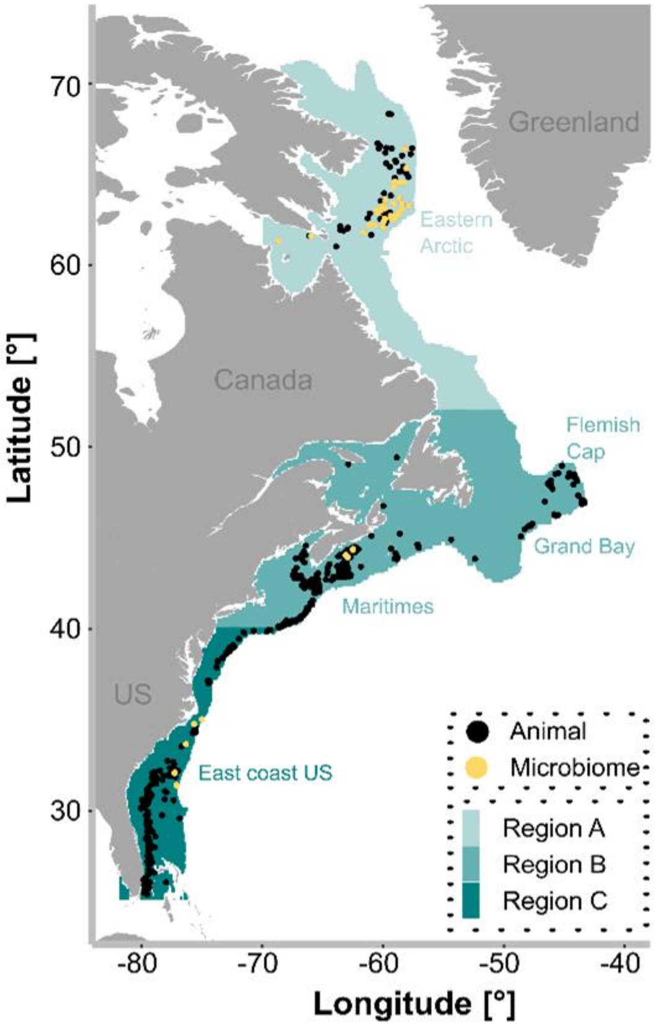

Supplementary Figure 1 Overview of study area, divided into three regions: Region A, Region B, and Region C. Animal and microbe occurrences used for analyses of this study are indicated by black and yellow dots, respectively.

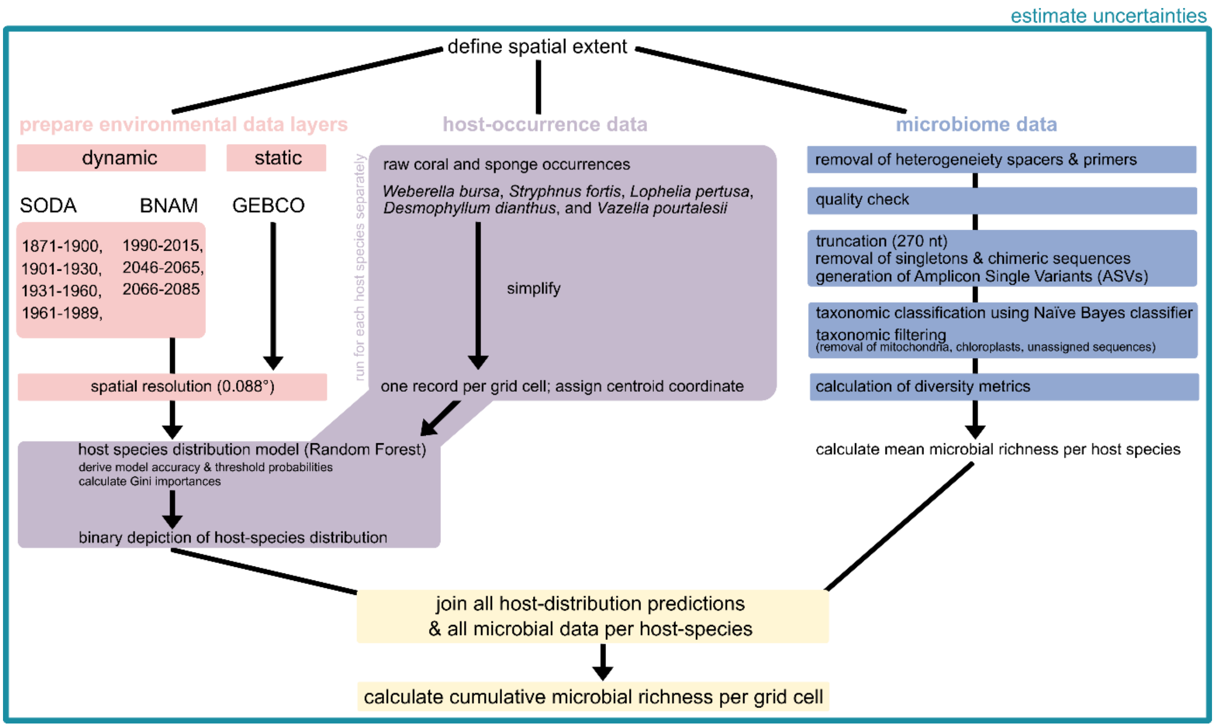

Supplementary Figure 2 Overview of detailed methodological workflow conducted for the identification of coral- and sponge-associated microbial biodiversity hotspots under past, present, and future scenarios.

	Time frame	Environmental dataset
Past	1871-1900	SODA
	1901-1930	SODA
	1931-1960	SODA
	1961-1989	SODA
Present	1990-2015	BNAM
Future	2046-2065	BNAM (RCP8.5)
	2066-2085	BNAM (RCP8.5)

Supplementary Figure 3 Overview of analysed time frames and environmental datasets used in this study.

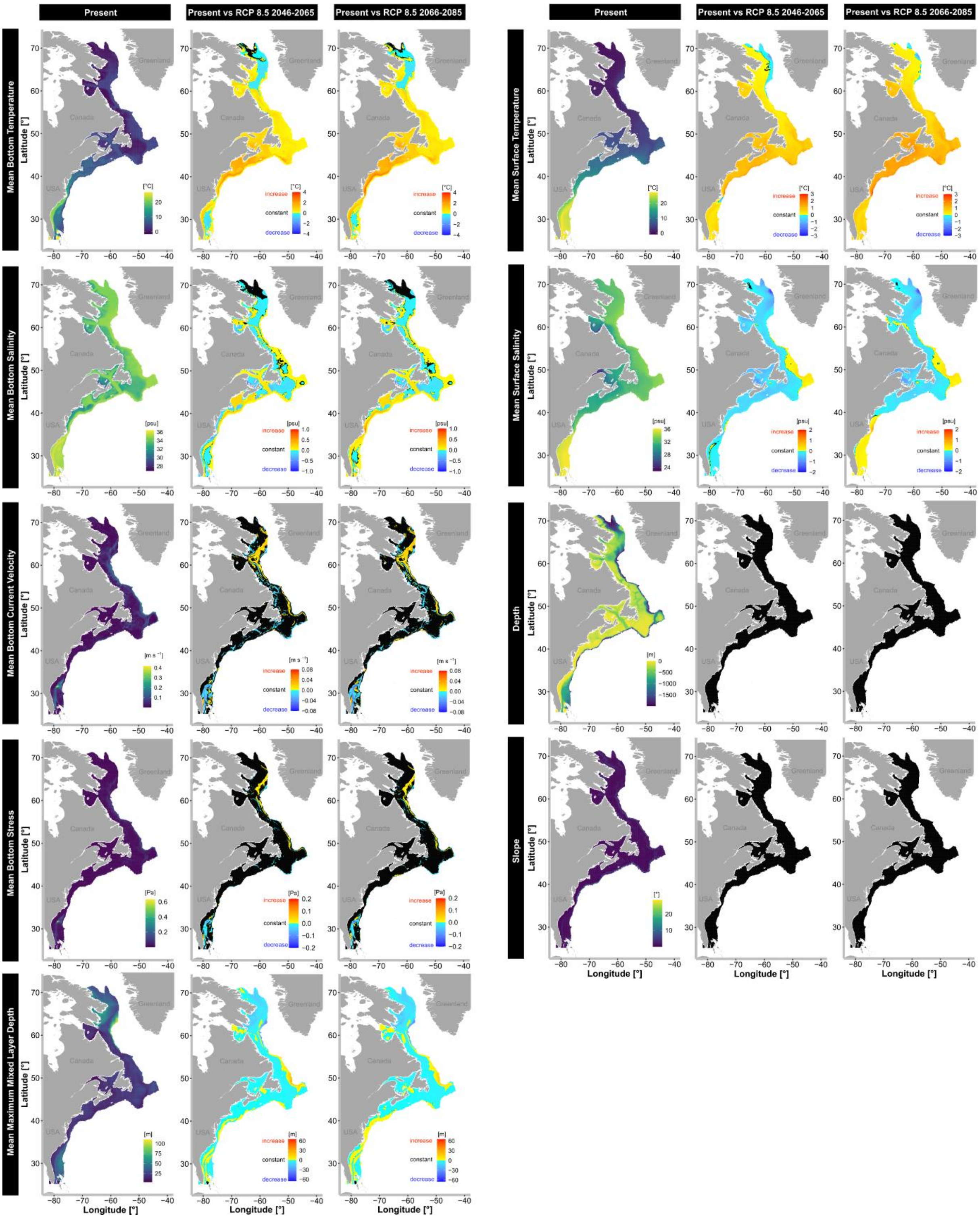

Supplementary Figure 4 Nine environmental parameters used for species distribution modelling for the present and two future timeframes. Oceanographic parameters were derived from BNAM. Maps for the present are given as absolute values, while the two future timeframes are composed delta-vales, showing differences in comparison to the present.

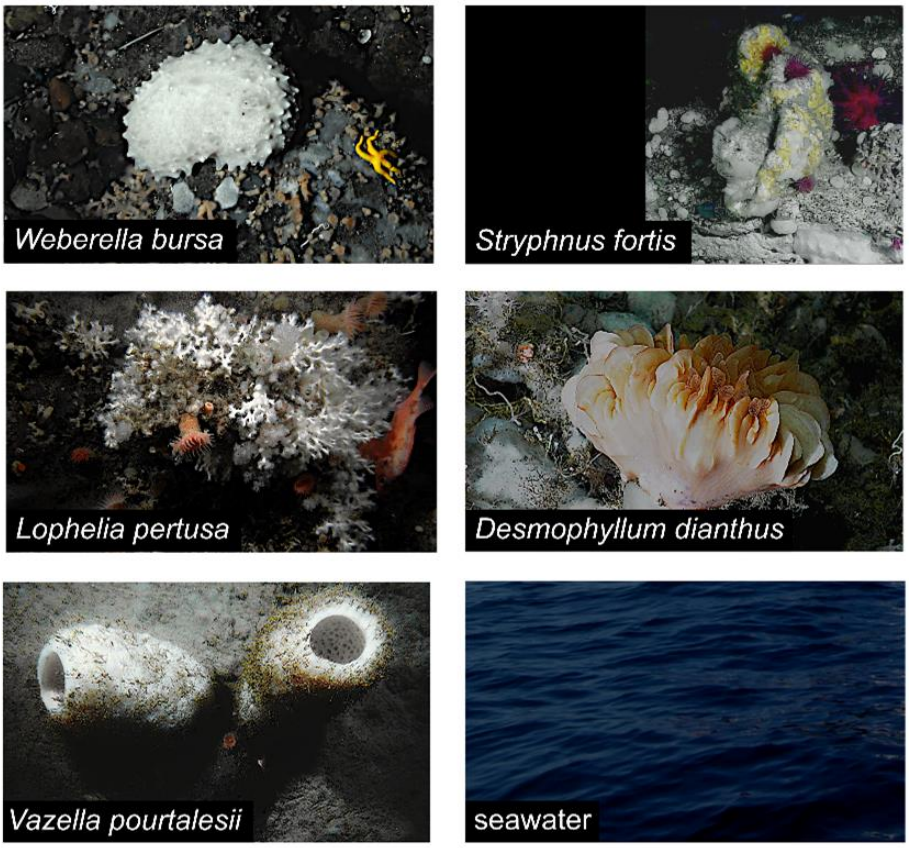

Supplementary Figure 5 Underwater photographs of study animal species *Weberella bursa*, *Stryphnus fortis*, *Lophelia pertusa*, *Desmophyllum dianthus*, and *Vazella pourtalesii*, as well as seawater reference.

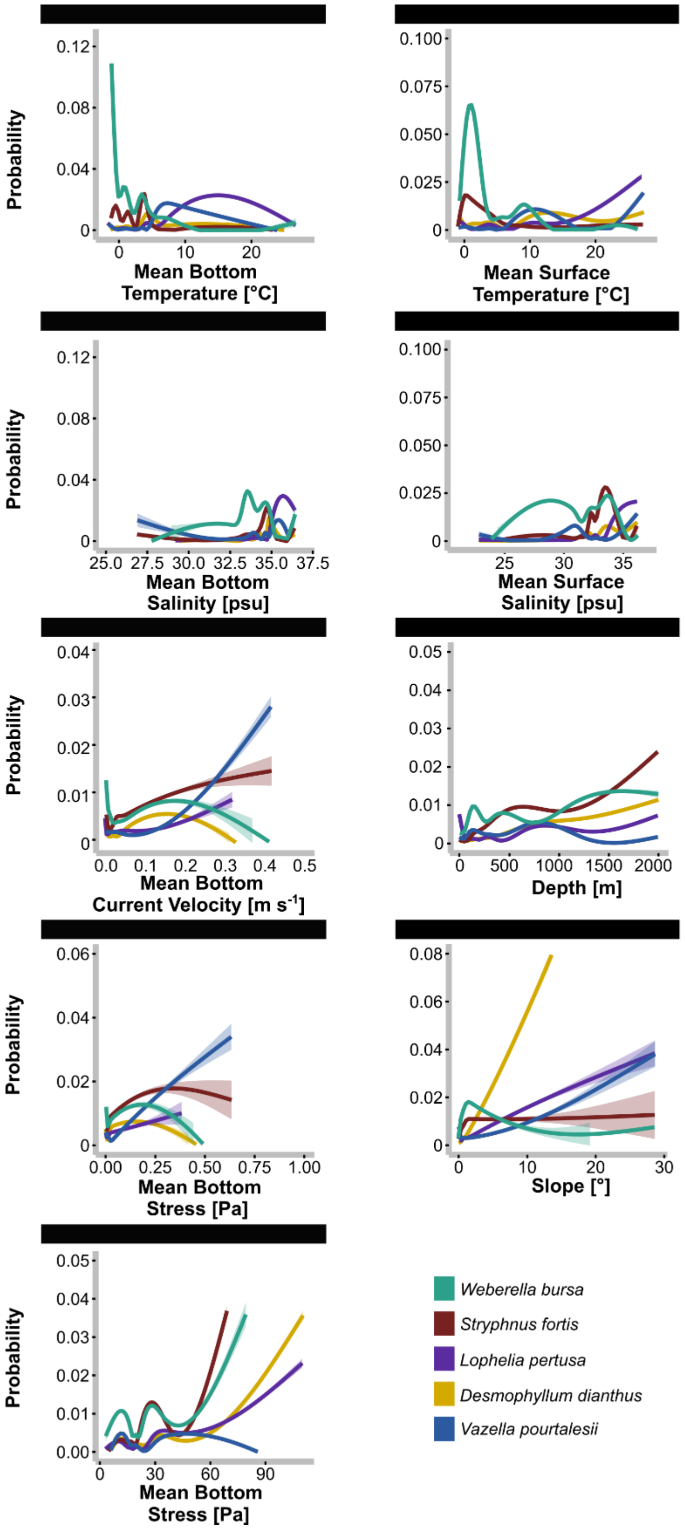

Supplementary Figure 6 Functional response curves, showing probabilities of animal occurrence for the five different species *Weberella bursa* (green), *Stryphnus fortis* (red), *Lophelia pertusa* (purple*),* *Desmophyllum dianthus* (yellow), and *Vazella pourtalesii* (blue) at different magnitudes of the nine evaluated environmental parameters. Approximate 95% confidence intervals are indicated by ribbons.

Supplementary Table 1 Summed predicted occupied area [km²] per host species across the seven different time frames.

Year	***Desmophyllum dianthus***	***Lophelia pertusa***	***Stryphnus fortis***	***Vazella pourtalesii***	***Weberella bursa***
1871 - 1900	1,926,005	1,855,677	636,157	945,122	1,834,066
1901 - 1930	1,912,042	1,827,308	632,242	897,097	1,837,189
1931 - 1960	1,872,229	1,797,215	655,113	901,840	1,799,432
1961 - 1989	1,876,922	1,791,341	655,889	891,873	1,810,119
1990 - 2015	252,847	217,412	312,258	175,161	562,537
2046 - 2065	550,787	518,704	327,759	474,057	823,030
2066 - 2085	615,282	512,827	335,448	469,983	778,308

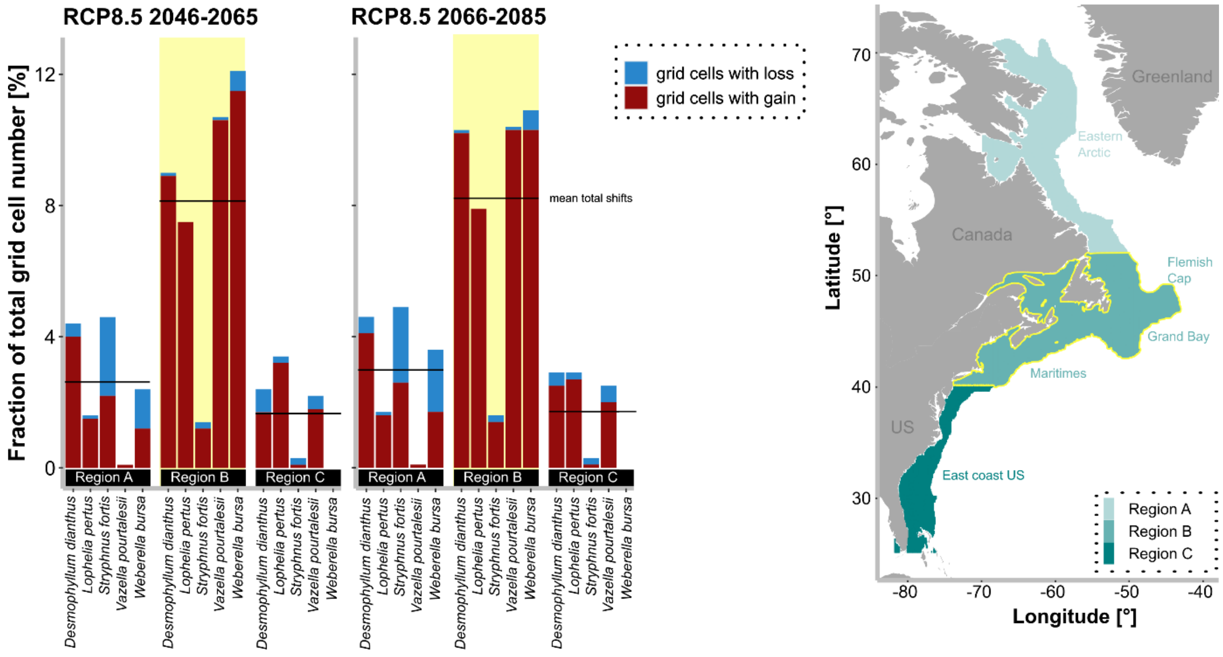

Supplementary Figure 7 Predicted future shifts in cumulative microbial richness in deep-sea corals and sponges across the three sub-regions (A, B, and C) indicated for the five coral and sponge species, given as fraction of total grid cell number. Mean total shifts are indicated by lines. As sub-region B depicts the largest shifts, it is highlighted in yellow within the barplot, but also on the map next to it.

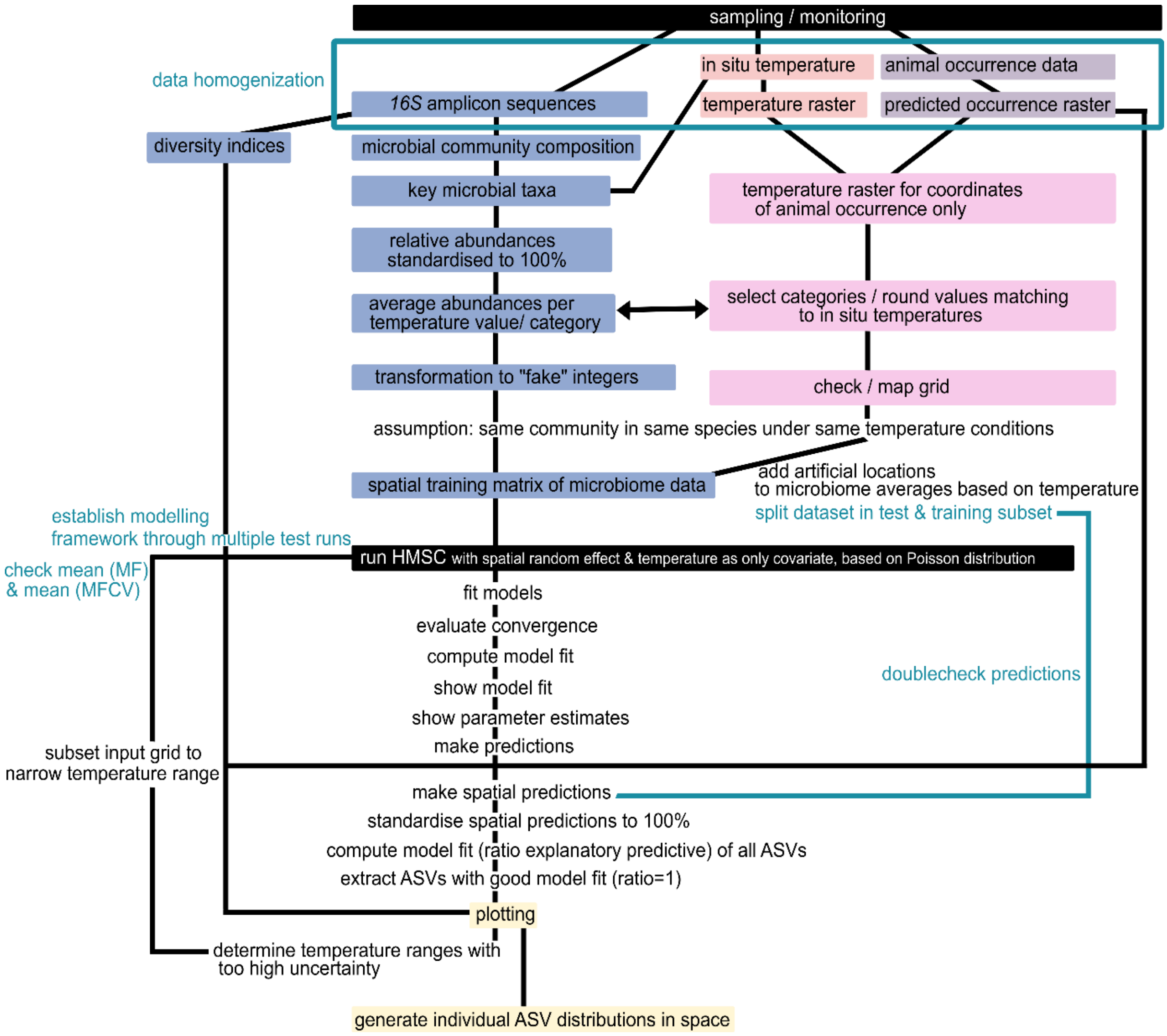

Supplementary Figure 8 Overview of detailed methodological workflow conducted for the prediction of variability in key microbial composition across an environmental gradient (temperature).

Supplementary Table 2 Overview of HMSC-model performance (explanatory power, predictive power, and ratio of explanatory power vs predictive power), as well as taxonomic names of the 15 key microbial taxa. Sometimes "bacteria" is abbreviated by "bac.", and "Candidatus" by "C."

**Name of ASV**	**Explana-tory**	**Predic-tive**	**Ratio**	**Phylum**	**Class**	**Order**
de44846a82b698f04dcc9cba350cbb8b	0.806	0.001	850.5	Proteobac.	Alphaproteobac.	
X88e0bc305eefd90b66bc9df365cd2f86	0.806	0.001	850.5			
X5fd878722e2825a002fb3459998d2f74	0.808	0.002	406.2	Proteobac.	Gammaproteobac.	
f25abba54a1a8042cfee90caae9eaac2	0.770	0.004	192.9	Proteobac.	Alphaproteobac.	Rhodospirillales
X4928b278e61381feca2dbec26ce8aa6c	0.770	0.017	45.4	Proteobac.	Alphaproteobac.	Rhodospirillales
a55e6860b2015dc7390a8e336e772f11	0.808	0.020	40.4	Proteobac.	Alphaproteobac.	
c2de3f4111059c39c137fc464ac1b60c	0.806	0.038	21.4	Proteobac.	Alphaproteobac.	Rhodospirillales
d62803e4b3ddeb077fafa7235cff329d	0.727	0.185	3.9	Patescibac.	Parcubacteria	C. Kaiserbacteria
X0b3da0a4e278462ae0fbc0c57572d068	0.771	0.218	3.5			
bcb979ea7d6db9f1e81929d5a490002c	0.678	0.352	1.9			
X016474f092254f68cbfe8b808fbdebe8	0.771	0.643	1.2	Patescibac.	Parcubacteria	C. Kaiserbacteria
X5087364294032ebd0b1a43a192c67ad9	0.572	-0.205	-2.8			
X4bb9b107c0e510457ce1a865308b76ea	0.572	-0.105	-5.4	Proteobac.	Alphaproteobac.	
a69ce95b29bec5ec5f35e338b1e2d547	0.807	-0.025	-32.7			
X7526df265f1117b77e0ee234926eebe7	0.807	-0.025	-32.7	Actinobac.	Acidimicrobiia	Microtrichales

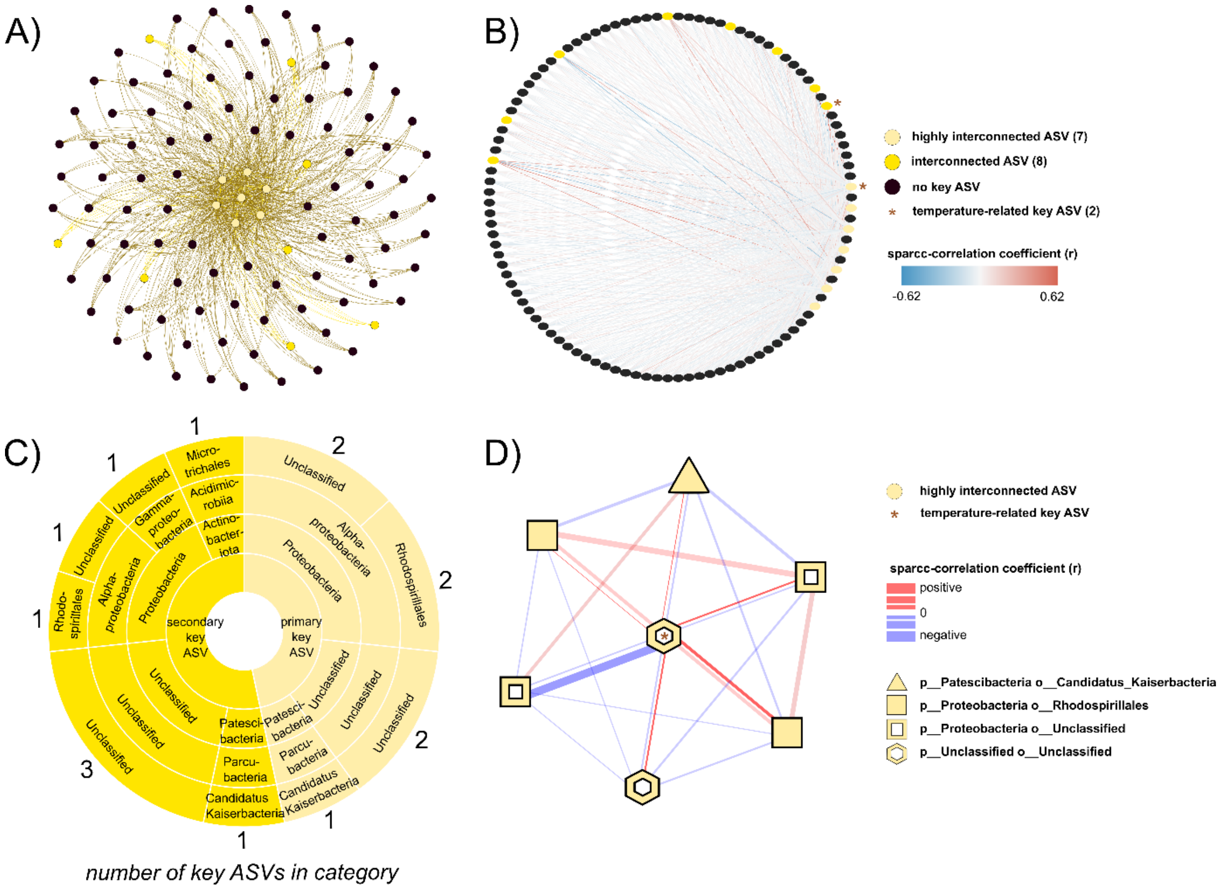

Supplementary Figure 9 A) Furchterman Reingold network presentation, highlighting seven key ASVs with particularly high network connections in the central part of the network. Those seven key ASVs are called “highly interconnected ASVs” in the following. Other key ASVs which have less network connections (and only to other key ASVs) are also indicated by yellow color, and called “interconnected ASVs” in the following. ASVs which have a connection with a key ASV, but are not key ASVs themselves, are marked by black color. **B)** Circular network presentation of the same network as in A). Directions of correlations between individual ASVs are marked by colors: blue=negative correlation, red=positive correlation. The same yellow and black color code is applied as in A). In addition, temperature-related key ASVs are marked by an asterisk. **C)** Sunburst diagram, showing taxonomic composition of highly interconnected and interconnected ASVs (phylum, class, and order-level). The number of key ASVs in each category is written at the outer part of the plot. **D)** Subnetwork in between highly interconnected ASVs. Symbols of nodes represent taxonomic classification of each ASV. The temperature-related key ASV is presented in the center of the network and highlighted with an asterisk.

Supplementary Table 3 Overview of microbial abundance status of key sponge species at the Flemish Cap. A-B= asexual-budding, BS=sexual broadcast spawner, BID=sexual brooder-indirect development pelagic, BDD=sexual brooder-direct development demersal or viviparous, PP=pelagic planktotrophic, PL= pelagic lecithotrophic, B= benthic.

**Taxa**	**Status**	**Longevity**	**Reproductive method**	**Propagule dispersal**
Asconema foliatum	LMA	> 50 years	BID	PL
Geodia barretti	HMA	> 50 years	A-B, BS	PL
Geodia macandrewii	HMA	> 50 years	A-B, BS	PL
Geodia parva-phlegraei	HMA	> 50 years	A-B, BS	PL
Mycale (Mycale) lingua	LMA	> 50 years	A-B, BID	PL
Stelletta normani	HMA	> 50 years	A-B, BS	PL
Stryphnus fortis	HMA	> 50 years	A-B, BS	PL
Stylocordyla borealis	LMA	> 50 years	BDD	B
Tentorium semisuberites	LMA	> 50 years	A-B, BS	PL
Weberella bursa	LMA	> 50 years	A-B, BS	PL

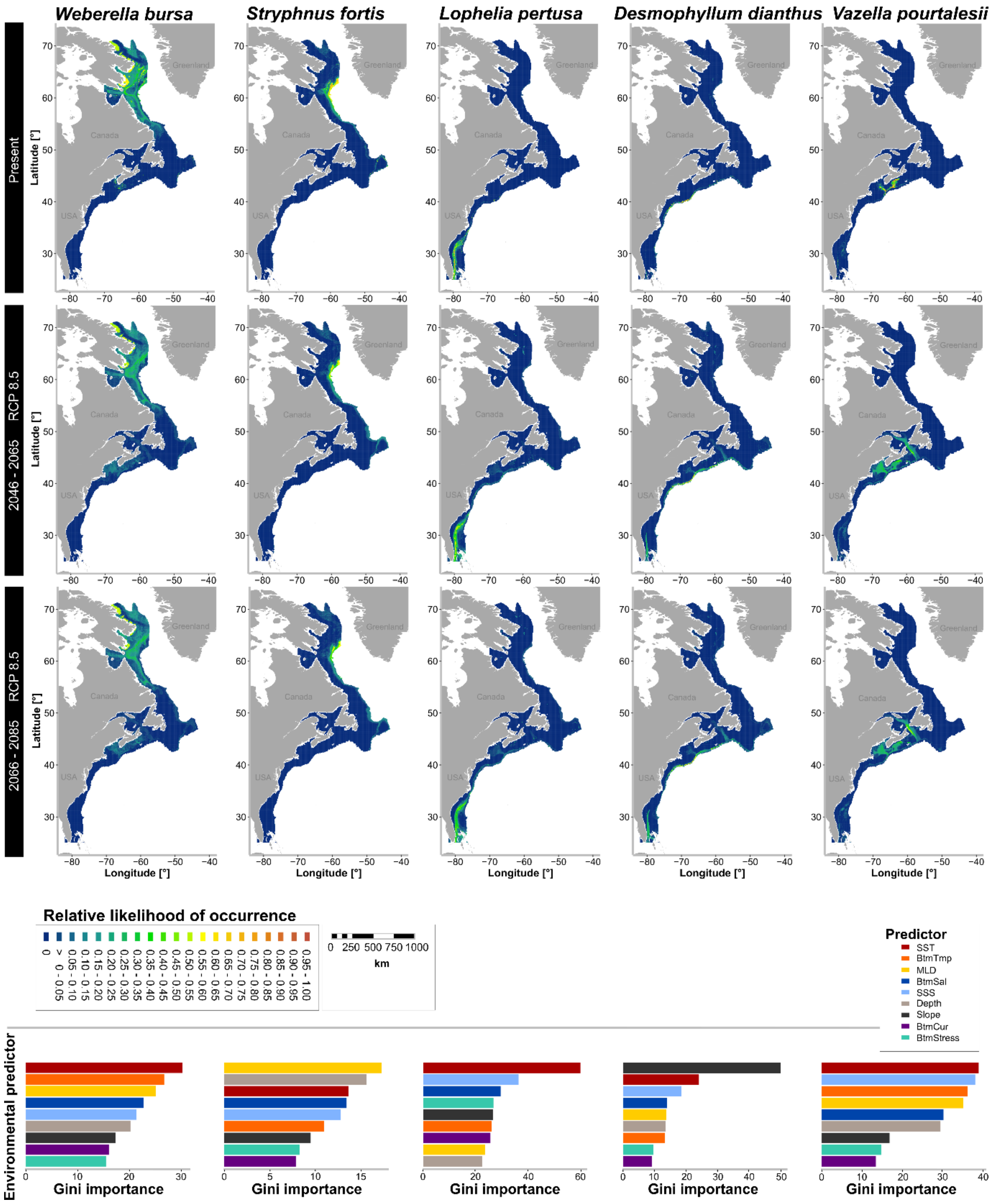

Supplementary Figure 10 Relative likelihood of occurrence of *Weberella bursa*, *Stryphnus fortis*, *Lophelia pertusa*, *Desmophyllum dianthus*, and *Vazella pourtalesii* from Random Forest predictions under present day and future (RCP8.5 2046-2065 and 2066-2085) climatic conditions. Gini importances (bar charts) indicate ranking of environmental predictor importance in Random Forest models for each animal species.

Supplementary Table 4 Accuracy measures for Random Forest models trained and tested on the *Weberella bursa, Stryphnus fortis, Lophelia pertusa, Desmophyllum dianthus*, and *Vazella pourtalesii* presence/pseudo-absence data of our study area. Cross-validation was done via 5-fold spatial blocking with random assignment of blocks into folds. Sensitivity, specificity, and the true skill statistic (TSS) were generated from a confusion matrix of tabulated outcomes that was thresholded using the maximum of sensitivity + specificity (MSS) identified each model. AUC: area under the receiver operating characteristic curve.

**Animal species**	**Mean AUC + - SD**	**Sensitivity + - SD**	**Specificity + - SD**	**TSS**	**MSS threshold**
Weberella bursa	0.83 +- 0.01	0.63 +- 0.05	0.87 +- 0	0.49	0.03
Stryphnus fortis	0.96 +- 0.01	0.93 +- 0.04	0.90 +- 0	0.83	0.02
Lophelia pertusa	0.95 +- 0.01	0.94 +- 0.02	0.89 +- 0	0.83	0.02
Desmophyllum dianthus	0.94 +- 0.00	0.92 +- 0.03	0.86 +- 0	0.78	0.01
Vazella pourtalesii	0.96 +- 0.01	0.95 +- 0.02	0.85 +- 0	0.80	0.02

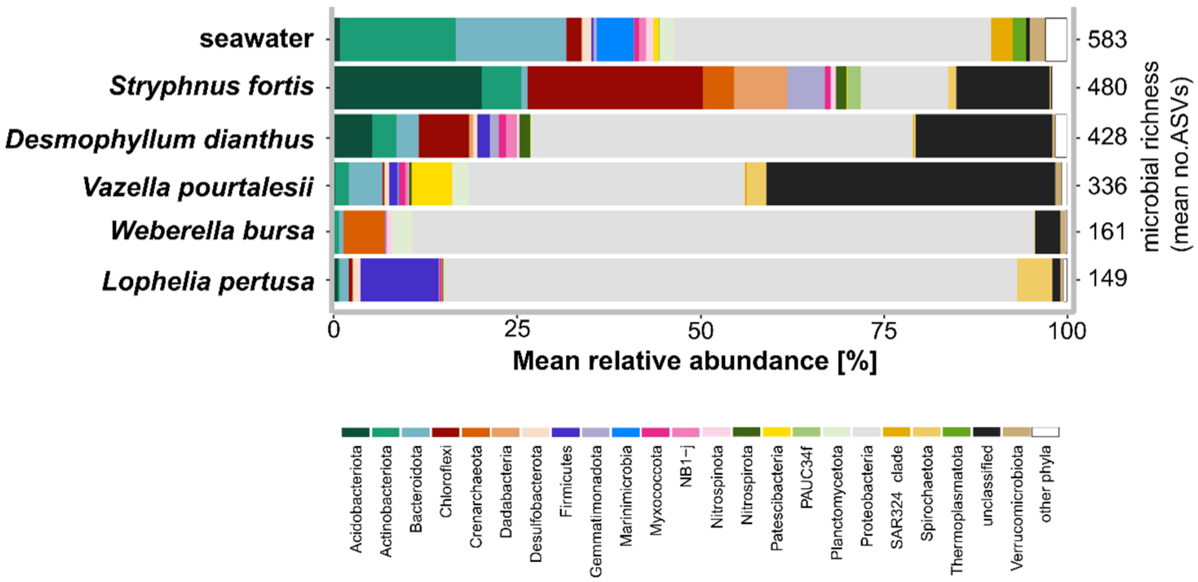

Supplementary Figure 11 Mean relative abundances of microbial phyla per sample type. Sample types are sorted after mean microbial richness, in descending order from top to bottom. Unclassified microbial taxa are marked by black bars. Standard error (SE) was calculated for mean microbial richness per host-species. It was highest in *Desmophyllum dianthus* (± 128 SE), followed in descending order by *Lophelia pertusa* (± 68 SE), *Stryphnus fortis* (± 33 SE), *Vazella pourtalesii* (± 26 SE), and *Weberella bursa* (± 17 SE).

Supplementary Table 5 Overview of mean standard deviations of Random-Forest predictions averaged across all grid cells. Means are shown per host species across all years, as well as sums of uncertainties across all host species per time frame.

**Year**	***Desmophyllum dianthus***	***Lophelia pertusa***	***Stryphnus fortis***	***Vazella pourtalesii***	***Weberella bursa***	***sum***
1871 - 1900	0.083	0.115	0.011	0.014	0.031	0.254
1901 - 1930	0.081	0.113	0.011	0.013	0.031	0.249
1931 - 1960	0.079	0.111	0.012	0.013	0.030	0.245
1961 - 1989	0.079	0.111	0.012	0.014	0.029	0.245
1990 - 2015	0.004	0.006	0.011	0.006	0.028	0.055
2046 - 2065	0.008	0.009	0.010	0.008	0.021	0.056
2066 - 2085	0.009	0.009	0.009	0.008	0.020	0.055
**mean**	0.049	0.068	0.011	0.011	0.027	

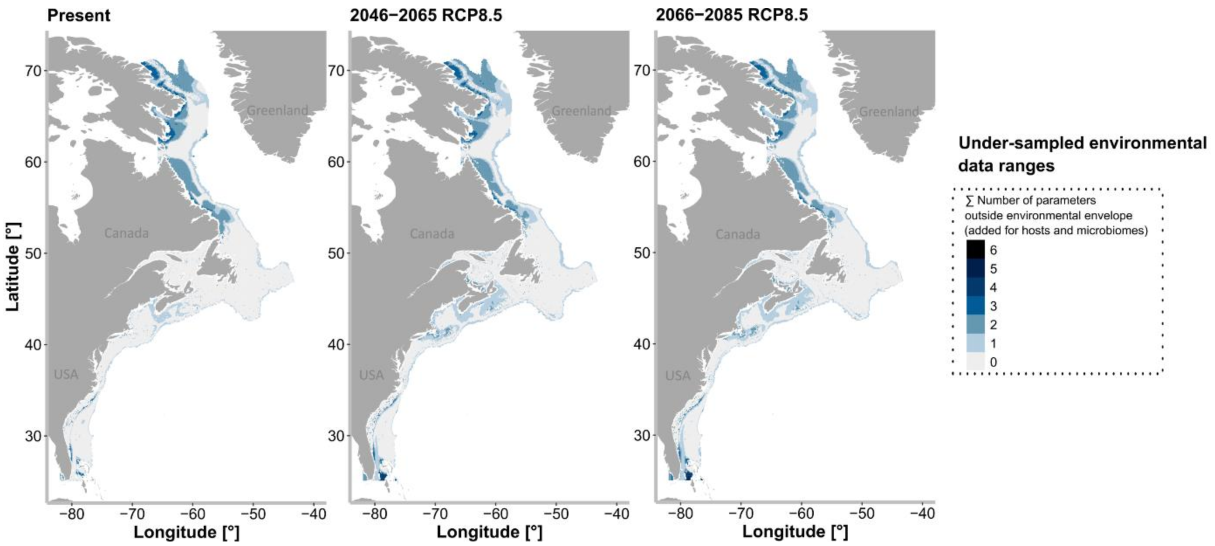

Supplementary Figure 12 Under-sampled environmental data ranges as an uncertainty source. The total numbers of parameters falling outside the actually sampled environmental envelope (added for hosts and microbiomes) are plotted per grid cell using the blue color gradient. The Present and two future timeframes are shown.

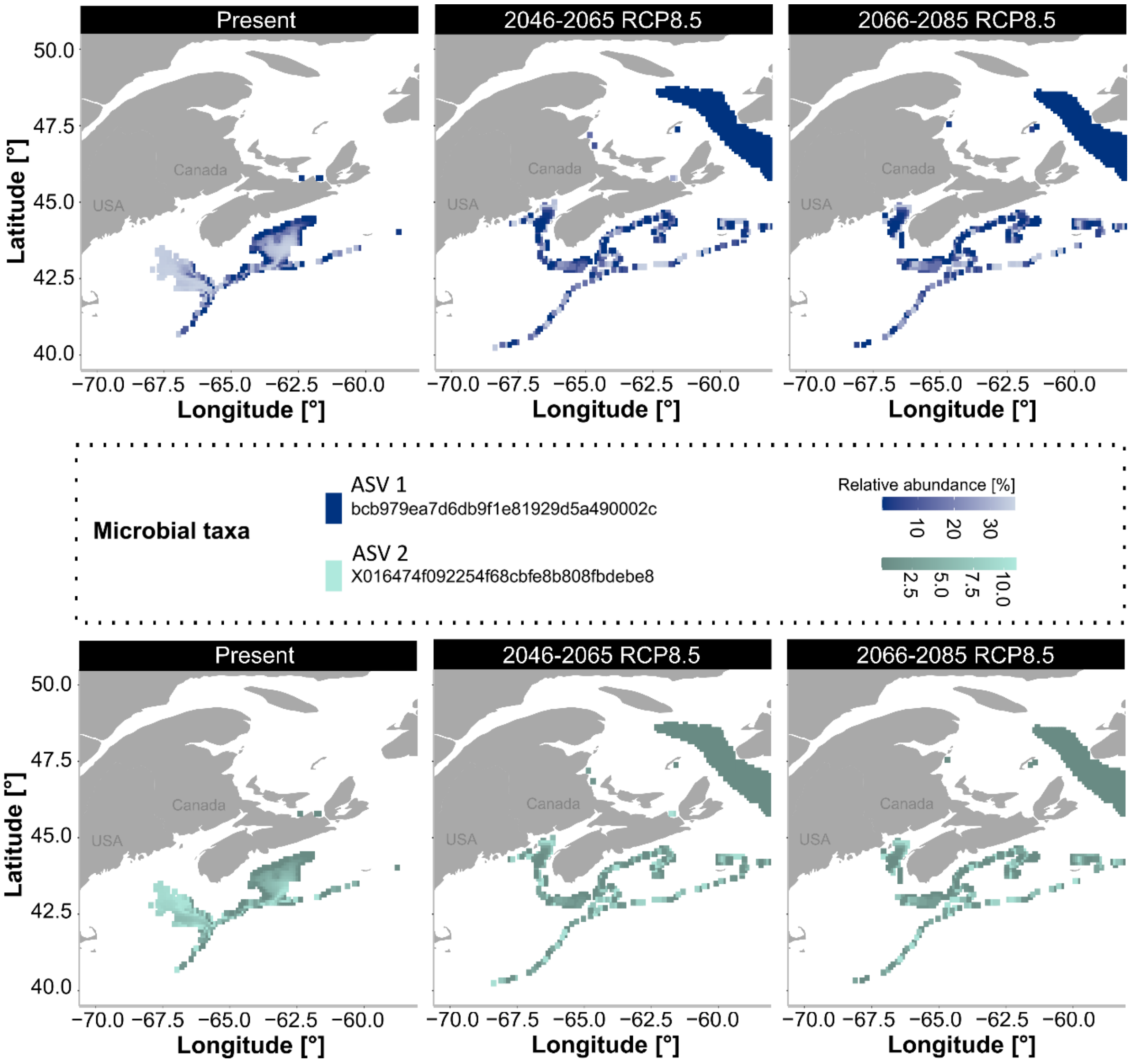

Supplementary Figure 13 Predicted distribution maps of relative abundances of key ASVs with good model fit in the glass sponge *Vazella pourtalesii* for the Present and two future timeframes. Two temperature-related key ASVs are shown by different colors. A temperature range of 6.2°C - 8.2°C is covered by the model. Areas with predicted occurrence of *Vazella pourtalesii* falling outside of this temperature range are not shown in the maps.

Supplementary Table 6 Accuracy measures for Random Forest models trained and tested on the HMA and LMA data of the Flemish Cap. Cross-validation was done via 5-fold spatial blocking with random assignment of blocks into folds. Sensitivity, specificity, and the true skill statistic (TSS) were generated from a confusion matrix of tabulated outcomes that was thresholded using the maximum of sensitivity + specificity (MSS) identified each model. AUC: area under the receiver operating characteristic curve.

**Host microbial abundance status**	**Mean AUC + - SD**	**Sensitivity + - SD**	**Specificity + - SD**	**TSS**	**MSS threshold**
HMA	0.76 +- 0.09	1.00 +- 0.00	0.60 +- 0.04	0.60	0.05
LMA	0.58 +- 0.03	0.64 +- 0.06	0.53 +- 0.05	0.17	0.44

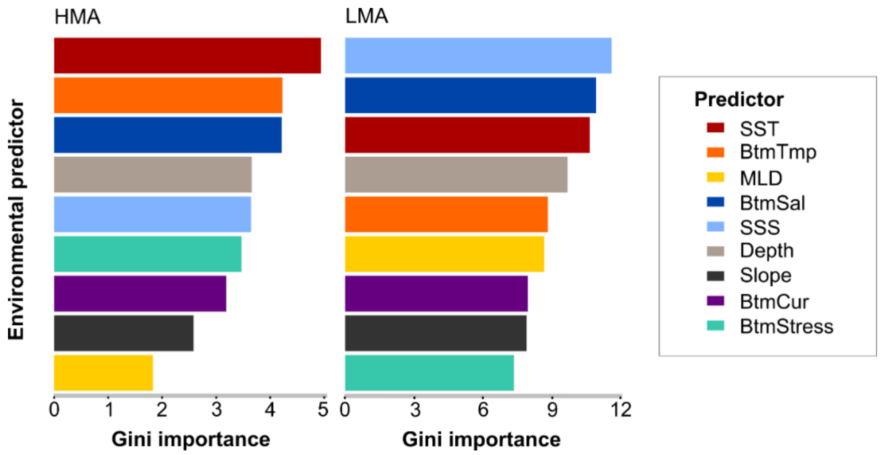

Supplementary Figure 14 Gini importances indicate ranking of environmental predictor importance in Random Forest models for each microbial abundance status.

Supplementary Table 7 Results of Spearman correlations between predictions of two overall ecosystem functions at the Flemish Cap (taken from [1]) and spatial predictions of sponge microbial abundance status occurrence.

	Nutrient cycling		Habitat provision	
**Group**	**Correlation coefficient**	**p-value**	**Correlation coefficient**	**p-value**
HMA	0.217	<0.001	0.463	<0.001
LMA	0.665	<0.001	0.589	<0.001

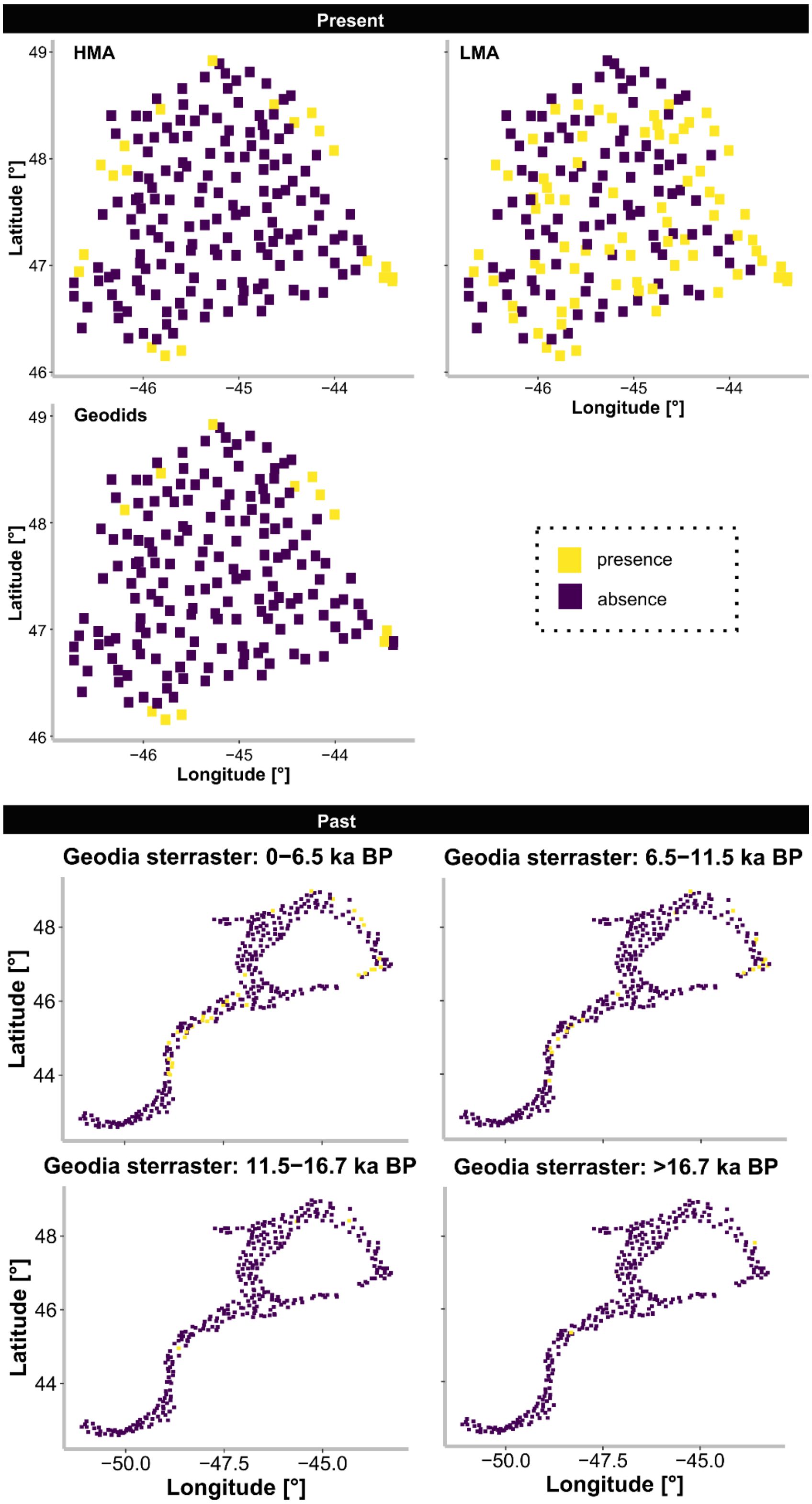

Supplementary Figure 15 Trait over time. **Upper panel:** Present raw occurrence data of the HMA-status, the LMA-status, and geodiids at the Flemish Cap. Data was taken from [1]. **Lower panel:** Records of *Geodia* sterrasters found in different age layers in cores taken at the Flemish Cap. Data was taken from[2]. Note that presence of sterrasters occurs at similar places from the past to the present.

References Supplementary Materials

1. Murillo, F. J., Weigel, B., Bouchard Marmen, M. & Kenchington, E. Marine epibenthic functional diversity on Flemish Cap (north-west Atlantic)—Identifying trait responses to the environment and mapping ecosystem functions. *Divers. Distrib.* **26**, 460–478 (2020).

2. Murillo, F. J., Kenchington, E., Lawson, J. M., Li, G. & Piper, D. J. W. Ancient deep-sea sponge grounds on the Flemish Cap and Grand Bank, northwest Atlantic. *Mar. Biol.* **163**, 1–11 (2016).
